# Shared Decision-Making (SDM) for Female SUI: Current Practice in Three Western Countries

**DOI:** 10.1007/s00192-025-06147-5

**Published:** 2025-04-17

**Authors:** Nienke J. E. Osse, Karine Gontijo-Santos Lima, Marian K. Engberts, Hugo W. F. van Eijndhoven, Wenche M. Klerkx, Michiel R. de Boer, Philippe D. Violette, Laura N. Nguyen, Rufus Cartwright, SDM in SUI Group, Maryam Sheikh, Maaike J. M. Pijpers, Jennifer Tang, Anna C. Verkleij-Hagoort, Marco H. Blanker, Paul. L. P. Brand

**Affiliations:** 1 Department of Primary- and Long-Term Care, University Medical Center Groningen, Hanzeplein 1, 9713 GZ Groningen, the Netherlands; 2 Department of Obstetrics and Gynaecology, Isala Hospital, Dokter Van Heesweg 2, 8025 AB Zwolle, the Netherlands; 3 Department of Gynaecology, St. Antonius Hospital, Soestwetering 1, 3543 AZ Utrecht, the Netherlands; 4 Departments of Surgery and Health Research Methods Evidence and Impast, McMaster University, 310 Juliana Dr, Woodstock, ON N4 V 0 A4 Canada; 5 Division of Urology, Department of Surgery, McMaster University, 1280 Main St W, Hamilton, ON L8S 4L8 Canada; 6 Department of Gynaecology, Chelsea and Westminster NHS Foundation Trust, 369 Fulham Rd, London, SW10 9 NH UK; 7 Department of Medical Education and Faculty Development, Isala Hospital, Dokter Van Heesweg 2, 8025 AB Zwolle, the Netherlands; 8 Wenckebach Institute for Medical Education, University Medical Center Groningen, Hanzeplein 1, 9713 GZ Groningen, the Netherlands

**Keywords:** Patient perceptions, Physician perceptions, Mixed, Methods study, Shared decision, Making, Stress urinary incontinence, Treatment decision

## Abstract

**Introduction:**

Different decision-making styles can be used to provide counselling for the multiple reasonable treatment options for patients with stress urinary incontinence (SUI). Shared decision-making (SDM) is currently advocated as the preferred style for preference sensitive decisions, as SDM takes patient preferences into account. This study aimed to map the current decision-making process for SUI in three Western countries.

**Methods:**

We included 124 patients and 18 physicians in a multicentre, prospective study in five hospitals in Canada, the United Kingdom and the Netherlands. We used patient and physician versions of the Control Preference Scale (CPS) questionnaires and examined audio-recordings of consultations with the OPTION-5 instrument to assess the degree of SDM.

**Results:**

Most patients (63%) perceived the decision-making as informative, some (29%) as shared and only a few (8%) as paternalistic. Dutch patients more often perceived the decision-making as informative than UK or Canadian patients. Patients’ preferred and perceived decision-making styles matched in 70% of consultations. Patients’ and physicians’ perceptions of decision-making were the same in 60% of consultations, but their perceptions of SDM use did not match. This also did not match the OPTION-5 scores reflecting the use of SDM. Almost all patients were satisfied with the decision-making they perceived.

**Conclusion:**

Most patients and physicians prefer and perceive the current decision-making process as informative decision-making. However, patients and physicians have different perceptions of their mutual consultation. This highlights the imprecise concept of SDM for both patients and physicians.

**Supplementary Information:**

The online version contains supplementary material available at 10.1007/s00192-025-06147-5.

## Introduction

Physicians provide counselling to patients with stress urinary incontinence (SUI) to make a decision for treatment that best suits the patient. Different approaches to decision-making can be used: the traditional paternalistic model in which the physician makes the decision based on medical knowledge, patient characteristics and evidence; the informative model in which the physician provides all the relevant information on treatment options and allows the patient to make the decision; and shared decision-making (SDM), in which decisions are made in a collaborative way between physician and patient, weighing both medical evidence and the patient’s views and preferences [1]. SDM is particularly advocated as the preferred model in preference sensitive decisions, i.e. cases with multiple reasonable treatment options, and enough time for patient and physician to thoroughly weigh those options [2]. Most patients prefer SDM, but many experience a discordance between their preferred decision-making style and how they perceive decision-making during consultation [3, 4].

The process of SDM has been operationalised into different steps or components, described in a commonly used four-step model [2]. In this model, the first step is informing the patient that a decision is to be made and that the patient’s opinion is important. The second step is explaining the options to the patient by the physician, with the pros and cons of each relevant option. In the third step, the patient and the physician discuss the patient’s preferences, and the physician supports the patient in the deliberation. In the final step, the physician and patient discuss the patients’ decisional role preference, make or defer the decision, and discuss possible follow-up [2]. Many different measures for decision-making exist, most of which are based on these steps [5–7].

Because of the availability of multiple reasonable treatment options, including no treatment, SUI is a textbook example of a medical problem suitable for SDM. A recent study among Dutch women with SUI indeed showed their strong preference for SDM [8]. It demonstrated their need to be provided with information on their disorder and treatment options, both for personalized health care and for consideration of their social context [8]. To date, however, no studies have examined what decision-making models are being used in clinical practice for patients with SUI, and to what extent these are in concordance with the patient’s preferred decision-making model [9].

Owing to the controversies in treatment with mid-urethral mesh slings in the UK, the treatment options for SUI differ between countries [10, 11]. The present study was conducted in three countries to allow a comprehensive overview of the current decision-making process for treatment of SUI. We explored patients’ preferences regarding decision-making styles, and their physicians’ perceptions of the current decision-making process. We established whether or not patients’ perceptions of the decision-making were in line with their preferences.

## Methods

### Study Design

We performed an international, multicentre, prospective study of women visiting their gynaecologist or urologist for the treatment of SUI. Patients were included from five general and university hospitals in Canada, the United Kingdom and the Netherlands. We studied the initial consultation about SUI, using patient questionnaires before and after the consultation, audio-recordings of the consultation, and a physician questionnaire after the consultation.

### Participants

Women between 18 and 80 years of age with pure SUI or stress-predominant mixed urinary incontinence (MUI), for whom first line treatment with pelvic floor muscle therapy was insufficient, were eligible for inclusion in this study. Exclusion criteria were prior incontinence or prolapse surgery, current pregnancy or desire for future pregnancy and cognitive impairment.

Physicians were eligible if they worked in one of the five participating hospitals and expected to counsel at least 10 patients for treatment with SUI during the course of the study.

Between May 2023 and August 2024, all consecutive eligible patients of a participating physician were approached for participation in the study by members of their healthcare team. Upon showing interest in participation, a member of the research team contacted them to provide more information and answer questions, after which patients provided written informed consent for completing two questionnaires and audio-recording of the consultation. Eligible physicians were provided with information on the study, after which they provided written informed consent for the questionnaire and audio-recording of the consultation.

### Questionnaires

Details of the English questionnaires used can be found in Appendices 1–3. In the Netherlands, the Dutch version of the (validated) questionnaires were used. The questionnaires were piloted and iteratively modified to optimize clarity. The pre-consultation patient questionnaire comprised demographic information and questions about SUI severity and impact, using the Sandvik Incontinence Severity Index (ISI) and the Patient Global Impression of Severity (PGI-S), and the Control Preference Scale (CPS), a validated instrument to assess the patient’s preferences in the decision-making process with five different response options [7, 12, 13]. The post-consultation questionnaire included an adapted version of the CPS, in which the patient’s perception of the decision-making process in the consultation was assessed (pCPS) [4]. Both the CPS and pCPS were recoded to three categories of decision-making: answer A and B were combined into informative decision-making, answer C was renamed as SDM, and answer D and E were combined into paternalistic decision-making. Patients also completed the 9-Item Shared Decision-Making Questionnaire (SDM-Q- 9) assessing the perceived use of SDM during the consultation [14]. We used the transformed score ranging from 0, the lowest possible level of SDM, to 100, the highest possible level of SDM. Finally, we assessed patient’s satisfaction with the consultation and the likelihood they would recommend this physician to a loved one on VAS-scales ranging from 0 to 10 [15]. Scores ≥ 8 were considered to represent satisfaction with the consultation and a recommendation of the physician, respectively.

The post-consultation questionnaire for physicians assessed demographic information and their perception of the decision-making process in the consultation (pCPS-DOC) [4]. The pCPS-DOC was recoded in the same manner as the CPS and pCPS. In addition, physicians completed an adapted version of the SDM-Q- 9 to assess their self-reported use of SDM, i.e. the Shared Decision-Making Questionnaire-Physician Version (SDM-Q-DOC) [14]. The SDM-Q-DOC was also transformed to a score ranging from 0 to 100.

All consultations in which a patient received counselling for SUI treatment and in which a treatment decision was made were audio-recorded. These audio recordings were analysed with the validated Observing Patient Involvement (OPTION- 5) instrument to assess the extent to which the participating medical specialists involved patients in the decision-making process regarding the treatment of their SUI (Appendix 4) [16]. Each OPTION- 5 item is scored on a 5-point Likert scale ranging from zero (not observed) to four (executed to a high standard). The sum of these items is the total score (range 0–20). Following the instrument’s coding manual, we rescaled the total scores to a range of 0–100 [17]. After two independent researchers scored the first consultation, the scores were compared and consensus was reached on all items. Subsequently, the first hundred consultations were independently scored by two researchers, the remaining consultations were scored by the first author only.

### Outcome Measures

The primary outcome was to determine patients’ perceived decision-making style, measured with the pCPS.

The secondary outcomes were determining patients’ preferred decision-making style (measured with the CPS) and physicians’ perceived decision-making style (measured with the pCPS-DOC), as well as establishing patients’ satisfaction with the consultation and patients’ recommendation of their physician.

We were interested in both the degree of agreement between preferred and perceived decision-making styles for patients (CPS vs. pCPS), and the degree of agreement between perceived decision-making styles for patients and physicians (pCPS vs. pCPS-DOC), and in differences between countries in these associations. We were also interested in the degree of agreement between perceived use of SDM for patients and physicians (SDM-Q- 9 vs. SDM-Q-DOC).

We assessed the associations between perceived decision-making style and perceived use of SDM for patients and physicians (pCPS vs. SDM-Q- 9 and pCPS-DOC vs. SDM-Q-DOC, respectively), as well as differences between countries in these associations. We also assessed the associations between the perceived use of SDM for the independent observer and the perceived decision-making style for both patients and physicians separately (OPTION- 5 vs. pCPS and OPTION- 5-DOC vs. pCPS-DOC, respectively). In addition, we assessed the associations between the perceived use of SDM for the independent observer and the perceived use of SDM for both patients and physicians separately (OPTION- 5 vs. SDM-Q- 9 and OPTION- 5-DOC vs. SDM-Q-DOC, respectively).

### Sample Size

In the absence of previous research on the decision-making process in specialist consultations for SUI, no sensible sample size calculation could be made. In accordance with earlier research on decision-making in medical specialist consultations, we aimed to include 10 patients per participating physician [4, 18].

### Statistical Analyses

Normally distributed variables were analysed with standard parametric statistical methods, and non-normally distributed variables with nonparametric methods.

Primary and secondary outcomes were described for the total population and compared between countries.

We assessed the degree of agreement between patients’ preferred and perceived decision-making style (CPS and pCPS), and between patients’ and physicians’ perceived decision-making style (pCPS and pCPS-DOC) using Sankey plots. We assessed patients’ and physicians’ perceptions of the use of SDM (SDM-Q- 9 and SDM-Q-DOC) with a Bland–Altman plot.

Owing to the small patient numbers in the paternalistic decision-making categories, the analyses on preferred and perceived decision-making styles were limited to the informative and shared decision-making categories.

Degree of similarity between the two independent observers scores of the OPTION- 5 was analysed using intraclass correlation coefficient (ICC) in a two-way random model based on absolute agreement. An ICC of ≤ 0.5 was labelled as poor agreement, 0.51–0.80 as average, 0.81–0.90 as good and 0.91–1.00 as excellent agreement. As we found good agreement between the observers, the observations of the first author were used in the further analyses.

Owing to the nested nature of the data, with multiple patients consulting the same physician, multilevel analyses were used to assess the associations between outcomes. These analyses involved two levels: the patient level (level 1) and the physician level (level 2), with a random intercept at the physician level. We used multilevel logistic regression analyses for the associations between patients’ perceived decision-making style (pCPS) for the different countries. We also used multilevel linear regression for the associations between patients’ perceived decision-making style and use of SDM (pCPS vs.and SDM-Q- 9), between physicians’ perceived decision-making style and use of SDM (pCPS-DOC vs. SDM-Q-DOC), between the observer’s assessment of use of SDM and patients’ perceptions of both decision-making style and use of SDM (OPTION- 5 vs. pCPS and OPTION- 5 vs. SDM-Q- 9, respectively), and between the observer’s assessment of use of SDM and physicians’ perceptions of both decision-making style and use of SDM (OPTION- 5 vs. pCPS-DOC and OPTION- 5 vs. SDM-Q-DOC, respectively).

The patient’s satisfaction with the consultation and the patients’ recommendation of the physician were analysed using descriptive statistics. Owing to observed ceiling effects, no further statistical analyses were performed on these outcomes.

Statistical analyses were performed using SPSS Statistics for Windows: version 29.0 (SPSS Inc., Chicago, IL, USA). Two-sided *p* values < 0.05 were considered statistically significant.

### Ethical Approval

Ethical approval was provided in all three countries.

## Results

The demographic information for patients (*n* = 125) and physicians (*n* = 18) are presented in Tables 1 and 2, respectively.

**Table 1:** Patient demographics

Demographics	Total	The Netherlands	The United Kingdom	Canada
N	125	64	20	41
Age mean ± SD	50 ± 12	46 ± 10	51 ± 14	55 ± 11
Marital status *n* (%)				
Unmarried/no registered partner	40 (32.0)	22 (34.4)	10 (50.0)	8 (19.5)
Married/Registered partner	82 (65.6)	42 (65.6)	9 (45.0)	31 (75.6)
Widow	3 (2.4)	0 (0)	1 (5.0)	2 (4.9)
Ethnicity *n* (%)				
Caucasian descent	109 (87.2)	61 (95.3)	10 (50.0)	38 (92.7)
Mixed descent	4 (3.2)	1 (1.6)	2 (10.0)	1 (2.4)
Asian descent	4 (3.2)	1 (1.6)	2 (10.0)	1 (2.4)
African descent	3 (2.4)	0 (0)	2 (10.0)	1 (2.4)
Middle Eastern descent	2 (1.6)	1 (1.6)	1 (5.0)	0 (0)
* Hispanic descent*	2 (1.6)	0 (0)	2 (10.0)	0 (0)
* North African descent*	1 (0.8)	0 (0)	1 (5.0)	0 (0)
Highest level of education n (%)				
None	1 (0.8)	0 (0)	0 (0)	1 (2.4)
Primary education	1 (0.8)	0 (0)	0 (0)	1 (2.4)
Secondary education	30 (24.0)	14 (21.9)	0 (0)	16 (39.0)
Further education	31 (24.8)	15 (23.4)	6 (30.0)	10 (24.4)
University education	62 (49.6)	35 (54.7)	14 (70.0)	13 (31.7)
Current employment *n* (%)				
Full-time	41 (32.8)	14 (21.9)	5 (25.0)	22 (53.7)
Part-time	53 (42.4)	43 (67.2)	7 (35.0)	3 (7.3)
No employment	12 (9.6)	5 (7.8)	5 (25.0)	2 (4.9)
Retired	19 (15.2)	2 (3.1)	3 (15.0)	14 (34.1)
BMI mean ± SD	28.1 ± 5.4	26.5 ± 5.0	27.3 ± 5.5	30.9 ± 4.8
Smoking *n* (%)				
Yes	21 (16.8)	9 (14.1)	3 (15.0)	9 (22.0)
No	94 (75.2)	55 (85.9)	16 (80.0)	23 (56.1)
Quit smoking	10 (8.0)	0 (0)	1 (5.0)	9 (22.0)
Postmenopausal n (%)	46 (36.8)	15 (23.4)	9 (45.0)	22 (53.7)
Sexually active n (%)	93 (74.4)	54 (84.4)	6 (30.0)	33 (80.5)
Vaginal deliveries median (IQR)	2 (1–2)	2 (1–2)	2 (1–3)	2 (1–3)
Ceasarian sections median (IQR)	0 (0–0)	0 (0–0)	0 (0–0)	0 (0–1)
Type of incontinence *n* (%)				
SUI	77 (61.6)	41 (64.1)	12 (60.0)	24 (58.5)
MUI	48 (38.4)	23 (35.9)	8 (40.0)	17 (41.5)
Duration of urinary incontinence (years) median (IQR)	9 (4–12)	8 (5–14)	4 (2–11)	6 (2–12)
ISI score *n* (%)				
Mild	3 (2.4)	0 (0)	3 (15.0)	0 (0)
Moderate	53 (42.4)	33 (51.6)	9 (45.0)	11 (26.8)
Severe	45 (36.0)	24 (37.5)	3 (15.0)	18 (43.9)
Very severe	24 (19.2)	7 (10.9)	5 (25.0)	12 (29.3)
PGIS *n* (%)*				
Normal	4 (3.2)	3 (4.7)	0 (0)	1 (2.5)
Mild	27 (21.8)	13 (20.3)	7 (35.0)	7 (17.5)
Moderate	55 (44.4)	32 (50.0)	8 (40.0)	15 (37.5)
Severe	38 (30.6)	16 (25.0)	5 (25.0)	17 (42.5)
Previous treatments n (%)				
Lifestyle changes	37 (29.6)	7 (10.9)	9 (45.0)	21 (51.2)
Incontinence material	94 (75.2)	48 (75.0)	10 (50.0)	36 (87.8)
Tampon	17 (13.6)	12 (18.8)	1 (5.0)	4 (9.8)
Pessary	14 (11.2)	3 (4.7)	6 (30.0)	5 (12.2)
Other**	15 (12.0)	5 (7.8)	5 (25.0)	5 (12.2)

*BMI* Body Mass Index; *ISI* Incontinence Severity Index; *MUI* Mixed urinary incontinence; *PGI-S* Patient Global Impression of Severity; *SUI* Stress urinary incontinence

^*^ One (Canadian) patient missing

^**^ Other previous treatments consisted of different forms of pelvic floor exercises, homeopathy, medication and PTNS

**Table 2:** Physician demographics

Demographics	Total	The Netherlands	The United Kingdom	Canada	
N	18	7	6	5	
Age mean ± SD	44 ± 8	43 ± 7	47 ± 9	42 ± 8	
Gender *n* (%)					
Male	6 (33.3)	1 (14.3)	2 (33.3)	3 (60.0)	
Female	12 (70.6)	6 (85.7)	4 (66.7)	2 (40.0)	
Ethinicity *n* (%)					
Caucasian descent	12 (66.7)	7 (100.0)	3 (50.0)	2 (40.0)	
Asian descent	4 (22.2)	0 (0)	2 (33.3)	2 (40.0)	
African descent	1 (5.6)	0 (0)	1 (16.7)	0 (0)	
Middle Eastern descent	1 (5.6)	0 (0)	0 (0)	1 (20.0)	

### Preferred and Perceived Decision-Making Styles

**Fig. 1:**
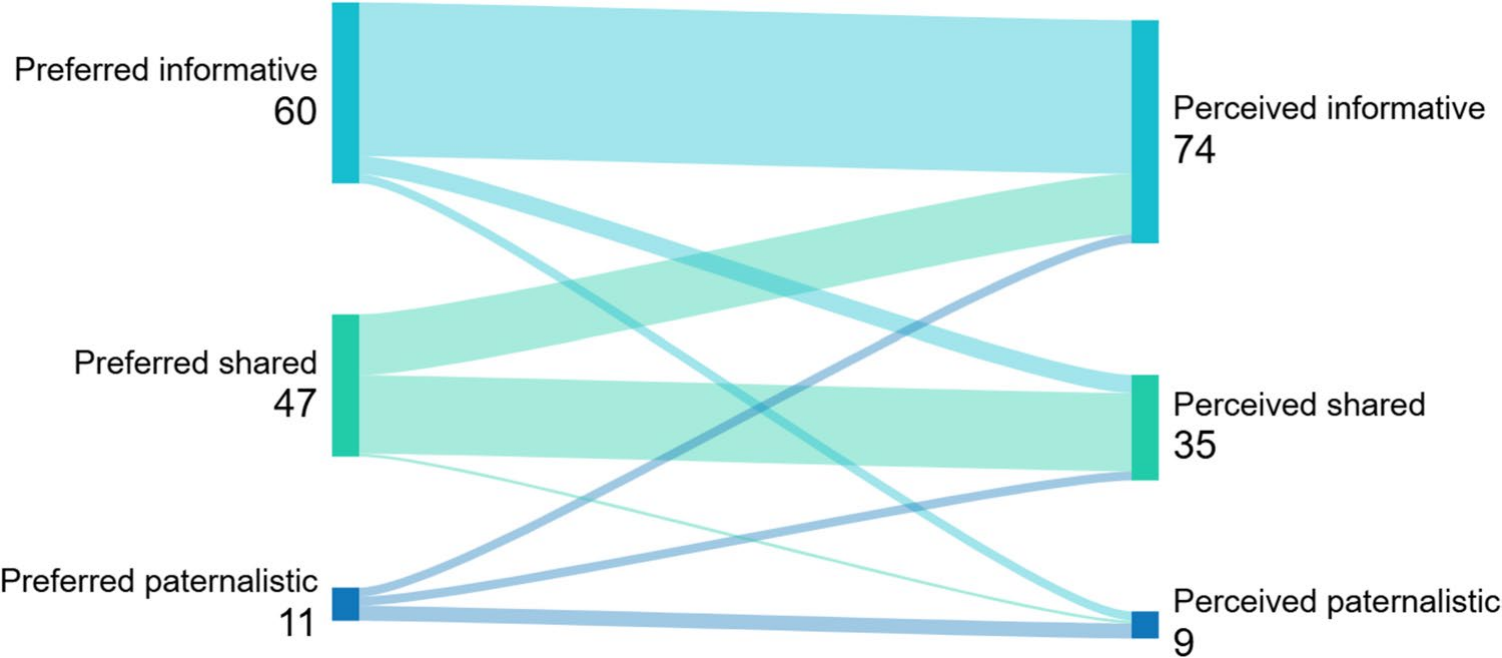
The degree of similarity between patients’ preferred and perceived decision-making styles (*n* = 118)

**Fig. 2:**
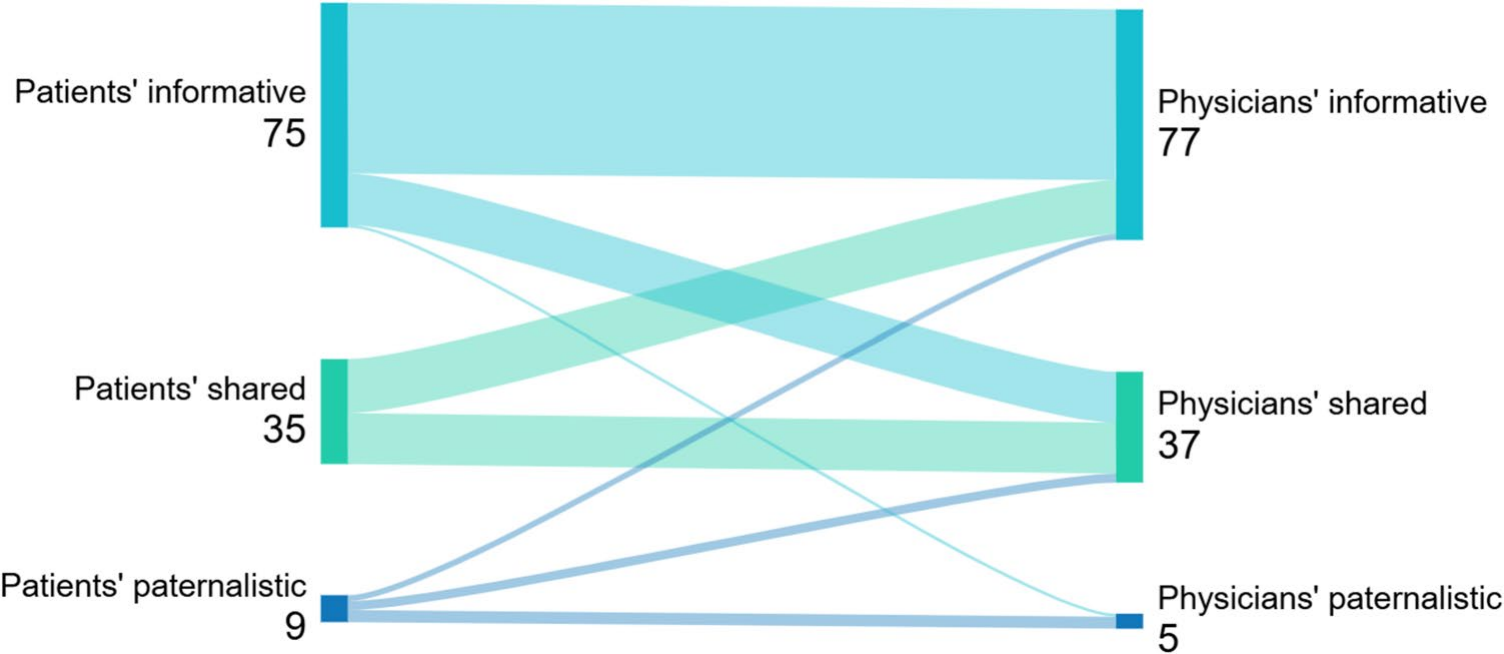
The degree of similarity between patients’ and physicians’ perceived decision-making styles (*n* = 119)

The preferred decision-making style of 124 patients was recorded, with 64 (52%) preferring informative decision-making, 49 (40%) preferring SDM and 11 (9%) preferring paternalistic decision-making. Of the 119 patients who recorded their perceived decision-making style, 75 (63%) perceived informative decision-making, 35 (29%) perceived SDM and 9 (8%) perceived paternalistic decision-making (Table 3, pCPS results). In 69% of consultations, patients’ preferred and perceived decision-making styles matched (Fig. 1). Figure 2 shows that patients’ and physicians’ perceived decision-making styles matched in 66% of the consultations. Considerable differences were found in the perceived decision-making style between the three countries (Table 3, pCPS results). The multilevel analysis of patients’ perceived decision-making styles showed an estimated proportion of 21% [95% CI 12;34] of Dutch patients perceiving SDM, compared to 65% [95% CI 36;85] for UK patients and 36% [95% CI 21;53] for Canadian patients.

**Table 3:** Patients perceived decision-making style per country

	Country			
*N* (% within country)	The Netherlands	The United Kingdom	Canada	Total *N* (%)
Perceived informative	45 (77.6)	5 (25.0)	25 (61.0)	75 (63.0)
Perceived shared	12 (20.7)	9 (45.0)	14 (34.1)	34 (29.4)
Perceived paternalistic	1 (1.7)	6 (30.0)	2 (4.9)	9 (7.6)
Total *N*	58	20	41	119

### Perceived use of SDM

**Fig. 3:**
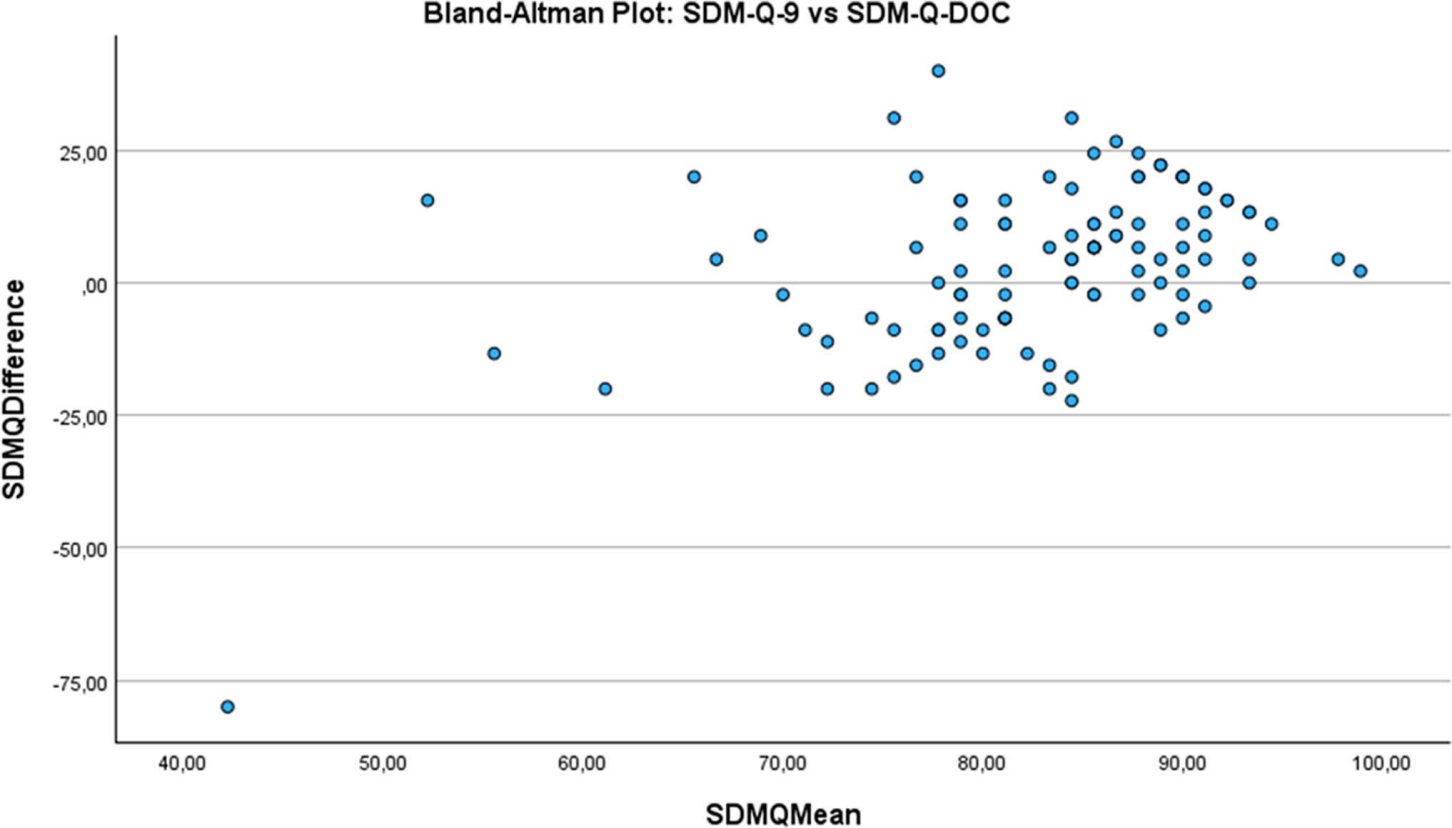
The degree of similarity between patients’ and physicians’ perceived use of shared decision-making (*n* = 108)

Table 4 shows the associations between patients’ and physicians’ perception of the decision-making process, and of SDM use, and the observers’ assessment of SDM use. Figure 3 presents patients’ and physicians’ perceived use of SDM, showing a mean difference in their SDM-Q score of 3.6 ± 15.4 (95% limits of agreement [− 26.5;33.6]).

**Table 4:** Use of shared decision-making for patients’ and physicians’ perceived decision-making style

pCPS scores	**N**	SDM-Q- 9 median (IQR)	**N**	OPTION- 5 mean ± sd
Perceived informative	68	86.7 (75.6–93.3)	74	44.4 ± 11.4
Perceived shared	31	91.1 (82.2–100.0)	33	38.6 ± 13.3
Perceived paternalistic	9	73.3 (63.3–88.9)	9	31.7 ± 15.6
Total	108	86.7 (75.6–95.0)	116	41.8 ± 12.8
**pCPS-DOC**	**N**	**SDM-Q-DOC median (IQR)**	**N**	**OPTION- 5 mean ± sd**
Perceived informative	73	82.2 (80.0–86.7)	79	44.9 ± 11.9
Perceived shared	35	80.0 (75.6–84.4)	38	38.9 ± 13.5
Perceived paternalistic	9	73.3 (58.9–83.3)	5	29.0 ± 8.9
Total	117	82.2 (77.8–86.7)	122	42.4 ± 12.8

*OPTION- 5* 5-item Observing Patient Involvement; *pCPS* Control Preference Scale perceptions version; *pCPS-DOC* Control Preference Scale physicians’ perceptions version; *SDM-Q- 9* 9-item Shared Decision-Making Questionnaire; *SDM-Q-DOC* Shared Decision-Making Questionnaire physicians’ version

Results of our multilevel analysis were inconclusive with respect to the association between the patients’ perceived use of SDM and their perceived decision-making styles (SDM-Q- 9 and pCPS), and with respect to differences between countries in perceived use of SDM. These multilevel analyses showed that patients that perceived informative decision-making had a mean difference in SDM-Q- 9 score of − 5.1 [95% CI − 10.9;0.8] compared to those who perceived SDM. In addition, estimated SDM-Q- 9 scores for British patients were lower and for Canadian patients higher compared to Dutch patients (− 3.4 [95% CI − 10.7;3.8] and 5.1 [95% CI − 0.9;11.2], respectively).

We found no evidence for an association between physicians’ perceived use of SDM and their perceived decision-making style (SDM-Q-DOC vs. pCPS-DOC), as physicians that perceived informative decision-making had a mean difference in SDM-Q-DOC score of 1.7 [95% CI − 1.2;4.5], compared to those that perceived SDM. The results of our multilevel analysis comparing SDM-Q-DOC scores between countries were inconclusive, showing lower estimated scores for British and similar scores for Canadian patients, compared to Dutch patients (− 7.2 [95% CI − 14.6;0.2] and 1.0 [95% CI − 6.4;8.4], respectively).

### Observer’s Assessment of the use of SDM

The ICC between the OPTION- 5 scores of the two independent researchers was 0.82 (95% CI 0.74–0.88), indicating good interobserver reliability. Table 4 shows the observer’s assessment of the use of SDM for each of the perceived decision-making categories. In multilevel analyses, we found similar estimated mean differences in OPTION- 5 scores for patients perceiving the decision-making as informative compared to SDM (5.3 [95% CI 0.9;9.6]) and for physicians perceiving the decision-making as informative, compared to those that perceived SDM (4.2 [95% CI − 0.5;8.9]), with wide and largely overlapping confidence intervals. For every ten points difference on the OPTION- 5, the estimated difference in SDM-Q- 9 was 0.7 points [95% CI − 0.9;2.2]. In contrast, for every ten points difference in OPTION- 5, the estimated difference in SDM-Q-DOC scores was 4.8 points [95% CI 2.4;7.3]. Compared to the Netherlands, British patients had a lower mean OPTION- 5 score, with a difference of − 15.0 [95% CI − 25.1;− 4.9], while Canadian patients did not seem to have a different mean OPTION- 5 score (− 5.5 [95% CI − 15.4;4.4]).

### Patient Satisfaction and Physician Recommendation Scores

Patient satisfaction scores ranged from 6 to 10, with a median score of 10 and only four patients scoring below 8. One patient scored a 0 on the recommendation score, commenting that she does not make these kinds of recommendations. After exclusion of this patient, the recommendation scores ranged from 7 to 10, with a median score of 10 and only three patients scoring below 8.

## Discussion

This study investigated the current treatment decision-making process in consultations for SUI. Almost all patients felt involved in the decision-making process, with more than half perceiving the decision-making as informative, a quarter as shared and only a few as paternalistic. Patients from Canada and the UK more often perceived SDM, while Dutch patients perceived more informative decision-making. Most patients perceived the decision-making style they preferred, and patients’ and physicians’ perceptions of the decision-making style commonly aligned. However, there was a mismatch between patients’ and physicians’ perceptions of the use of SDM. We also found little agreement between their perceptions of SDM and the independently observed physician’s use of SDM. Overall, patients were very satisfied with their consultation and would recommend their physician to others.

Most patients in our study perceived the decision-making as informative, while most patients with a range of medical problems in previous studies perceived SDM [4]. In contrast to a previous study on cancer patients, in which women perceived a passive role in consultations, the women in our study felt strongly involved in the consultation [19]. These findings align with those of a scoping review on the relationship between patient characteristics and SDM in treatment decisions, which reported associations in varying directions and strengths [20]. It is possible that our study population with a single condition and the mostly standardised counselling method for SUI made most patients feel they had the final say in the decision. The patients in this study were preselected after attempting pelvic floor physical therapy. As a result of limited success with conservative treatments, it is likely that both patients and physicians were more inclined to agree on pursuing surgery as the next step in treatment.

In line with findings from previous research, the patients’ preferred and perceived decision-making styles matched in almost 70% of consultations [4, 21]. Compared to a population of patients with a wide range of medical problems, our patient population wanted to be more involved in the decision-making (a preferred informative decision-making of 52% vs 15%) [4, 22]. A previous review showed that most patients want to be either involved in an informative or a shared way, but considerable differences were observed between conditions and patient populations [21, 23].

The differences we observed in perceived decision-making for SUI between the included Western countries are consistent with findings of cultural differences in urinary incontinence perceptions in a previous systematic review [24]. This review showed differences in perceptions regarding aetiology (non-white women expressed more self-blame of the symptoms), hygiene (Muslim women expressed difficulty with cleanliness requirements for religious obligations), and embarrassment (Hispanic women expressed more secrecy about their symptoms) [24]. Cultural differences also impact perceived decision-making in other conditions [25–27]. A scoping review on factors related to patients’ perceptions of SDM reported that restrictions within healthcare systems and their policies, differences in protocols guiding clinical decisions, financial constraints (e.g. insurance coverage), and the accessibility of healthcare were associated with patients’ perceptions of SDM [27]. Most SDM research to date has been conducted in the USA, Canada, the Netherlands and the UK, limiting an assessment of the role of cultural diversity on SDM perceptions [25]. Our findings on the differences between countries support earlier literature suggesting that cultural frameworks within healthcare systems significantly influence the success or challenges of implementing SDM. For example, in countries with more hierarchical healthcare systems, patients may expect doctors to make decisions on their behalf, hindering their active participation required for SDM. The British patients in this study were offered different treatment options compared to those in Canada and the Netherlands, as MUS surgery was not available in the UK. However, the outcomes of this study should not be impacted by this difference, as the focus was solely on the treatment decision-making process across various (surgical) treatment options, irrespective of the specific treatments offered.

In the current study, patients and physicians appeared to differ in their experience of the same consultation, predominantly in the distinction between informative and shared decision-making. Patients’ perceptions of decision-making were consistent with their own perceptions of the use of SDM. Patients are likely to feel involved in a consultation if they are able to ask questions, express their views, deliberate on options and come to an agreement with their doctor [28, 29]. It does not seem to matter whether they actually decide or delegate the decision to their doctor [29]. In addition, they feel more involved if their doctor draws attention to the fact that a decision needs to be made [23]. However, when comparing patients’ perceptions of SDM use with those of an observer, more SDM was observed in consultations that were perceived by patients as informative decision-making. Previous research also showed that patients and objective observers differ in their views of patient involvement [4]. A recent scoping review suggested that factors outside the communication between physician and patient, such as the availability of information about treatment options, time constraints and the involvement of other health care professionals can affect patients’ perceptions of the decision-making process in consultations with their physician [27]. This could also be the case for the patients in our study. In addition, multiple factors may come into play that could affect patient satisfaction with the consultation and the perceived decision-making, including decisional regret when the treatment outcome is worse than expected and outpatient clinic schedules running late because of organisational issues. As our investigation focussed on the decision-making processes within the consultation only, these factors and patient satisfaction after their treatment were not taken into account.

Physicians’ perceived decision-making style also showed a mismatch with their self-reported use of SDM and the assessment of the SDM process by an independent observer. Physicians who viewed their consultations as informative decision-making reported a similar use of SDM compared to those who perceived their consultations as SDM. This was also reported by an independent observer assessing the physician’s application of the steps in the SDM process. This is consistent with previous research showing that physicians cannot accurately assess their own decision-making style [30].

Almost all patients were satisfied with the consultation and would recommend their physician to others. In agreement with earlier research, these satisfaction and recommendation ratings were strongly skewed towards a ‘very satisfied’ response [31]. Although we used VAS scores in our satisfaction and recommendation assessments, which are less likely to be affected by confounding variables and ceiling effects than using a Likert scale, the ceiling effect of both these assessments precluded a meaningful analysis of their association with other outcome variables in the present study [31].

### Strengths and Limitations

To our knowledge, this was the first study researching the current treatment decision-making process in consultations for SUI. The main strength of this study was that it combines patient, physician and observer perceptions regarding the current treatment decision-making in consultations for SUI. The combination of these three views created a general overview of the current decision-making process. Even though both patients and doctors were aware of the study during the consultations, patients were not selected for this study by their physician. The use of validated methods and different perspectives supported the validity of our results.

The main limitations of this study were related to the instruments used. Firstly, during the application of the SDM-Q questionnaires, we anecdotally noted that the first item of the SDM questionnaire (stating ‘a decision needs to be made’), was misinterpreted as a ‘must’ by some patients and physicians, which may have affected their responses to the questionnaire. It is likely that this negatively affected the scoring of the perceived use of SDM. Secondly, the different instruments used in this study measured different aspects of the decision-making process, possibly measuring different constructs. This may have contributed to the discrepancies between the scores. Owing to logistical difficulties, not all included physicians were able to include the intended minimum of 10 patients. In addition, only a few patients reported a preferred or perceived paternalistic decision-making style, limiting the statistical power to analyse this decision-making style. However, as this was exploratory research, this study still provides insights into this previously unexplored topic.

The patients’ preferences for decision-making in this study differed from those reported in previous research in patients with various medical conditions [4]. Both this and previous studies showed limited cultural diversity in the patient population. This highlights the importance of exploring patients’ perspectives in different patient populations and different conditions. Patients seemed to perceive SDM as informative decision-making, which they prefer. This mismatch of terminology complicates the results of this study. To further support physicians in improving their counselling and decision-making, more insights should be sought in what aspects patients deem important in their treatment decision-making and how they feel the decision-making process could be further improved. Qualitative and mixed research methods are likely required to explore these issues further.

## Conclusion

Our study shows that most patients and physicians seem to perceive the current decision-making process for SUI in women as informative decision-making, and hardly any paternalistic decision-making is perceived. However, patients and physicians differ in their perceptions of the decision-making process in the same consultation. This shows that SDM remains an imprecise concept for both patients and physicians, allowing for multiple interpretations and highlighting the different perspectives on counselling and treatment decision-making. Overall, almost all patients are satisfied with the current decision-making process.

## Supplementary Information

Below is the link to the electronic supplementary material.

Supplementary file1 (DOCX 251 KB)

## Data Availability

Data will be made available upon request.

## Abbreviations

CPS: Control preference scale
ICC: Intraclass correlation coefficient
ISI: Incontinence severity index
OPTION- 5: Observing patient involvement – 5 items
PGI-S: Patient global impression of severity
pCPS: Control preference scale modified to perceptions
pCPS-DOC: Control preference scale modified to perceptions – physician version
SDM: Shared decision-making
SDM-Q- 9: Shared decision-making questionnaire – 9 items
SDM-Q-DOC: Shared decision-making questionnaire – physician version
SUI: Stress urinary incontinence
UK: United Kingdom
VAS: Visual analogue scale
