# Supplementary material for: Shared Decision-Making (SDM) for Female SUI: Current Practice in Three Western Countries
Source: Int Urogynecol J. 2025 Apr 17;36(10):1999–2009. doi: 10.1007/s00192-025-06147-5 (PMC12618321; doi:10.1007/s00192-025-06147-5)
Supplement: Supplementary file 1 — Supplementary file1 (DOCX 251 KB) [file 192_2025_6147_MOESM1_ESM.docx]

**Appendix 1** Patient questionnaire prior to the consultation

What is your marital status?

*Choose one of the options below*

- Not married/no registered partner
- Registered partner
- Married
- Widow

Which description of ethnicity would suit you best?

*Choose one of the options below*

- South Asian
- Chinese
- African/Black/Caribbean
- White/Caucasian
- Indigenous
- Latin/Latin American
- Other, namely…

Do you have a wish for future pregnancy/children?

- Yes, I (still) want to have children
- No, I don’t want to have (more) children

What is the highest level of education you finished?

*Choose one of the options below*

- Not finished education
- Primary education
- Secondary education
- Further education
- University
- A different education, namely…

What is your current employment?

*Choose one of the options below*

- Full-time
- Part-time
- No employment
- Retired

What is your weight?

What is your length/height?

Do you smoke?

- Yes
- No
- I quit

Are you postmenopausal (one year no menstruation)?

- Yes
- No

Are you sexually active?

- Yes
- No

How many vaginal deliveries did you have?

How many C-sections did you have?

How long have you had complaints of urinary incontinence?

Which treatments have you tried for your urinary loss?

*You can choose multiple options*

- Lifestyle changes
- Incontinence material (eg. pantyliners)
- Tampon
- Pessary
- Pelvic floor muscle therapy
- Surgery or bulkinjections
- Other, namely…

The following 3 questions are about your urinary incontinence.

*Check the one number that best describes how your urinary incontinence is now.*

| **1** | Normal |
| --- | --- |
| **2** | Mild |
| **3** | Moderate |
| **4** | Severe |

How often do you experience urinary leakage?

| **1** | Less than once a month |
| --- | --- |
| **2** | A few times a month |
| **3** | A few times a week |
| **4** | Every day and/or night |

How much urine do you lose each time?

| **1** | Drops |
| --- | --- |
| **2** | Small splashes |
| **3** | More |

The following question is about the upcoming consultation.

Which of the following forms of decision-making do you prefer regarding the choice for your treatment?

*Please choose a letter*

| **A** | I prefer to make the final decision about what treatment I will receive |
| --- | --- |
| **B** | I prefer to make the final decision of my treatment after seriously considering my doctor’s opinion |
| **C** | I prefer that my doctor and I share responsibility for deciding which treatment is best for me |
| **D** | I prefer that my doctor makes the final decision about which treatment will be used, but seriously considers my opinion |
| **E** | I prefer to leave all decisions regarding my treatment to my doctor |

**Appendix 2** Patient questionnaire after the consultation

Did you bring a companion to the consultation/was someone else present during the consultation?

- No
- Yes, who? (partner, parent, neighbour, etc)

What decision has been made during the consultation?

Which of the following forms of decision-making did you experience regarding the choice for your treatment?

*Please choose a letter*

| **A** | I made the final decision about what treatment I would receive |
| --- | --- |
| **B** | I made the final decision of my treatment after seriously considering my doctor’s opinion |
| **C** | My doctor and I shared responsibility for deciding which treatment is best for me |
| **D** | My doctor made the final decision about which treatment would be used, but seriously considered my opinion |
| **E** | My doctor made all decisions regarding my treatment |

Nine statements related to the decision-making in your consultation are listed below.

*For each statement please indicate how much you agree or disagree.*

1. My doctor made clear that a decision needs to be made.


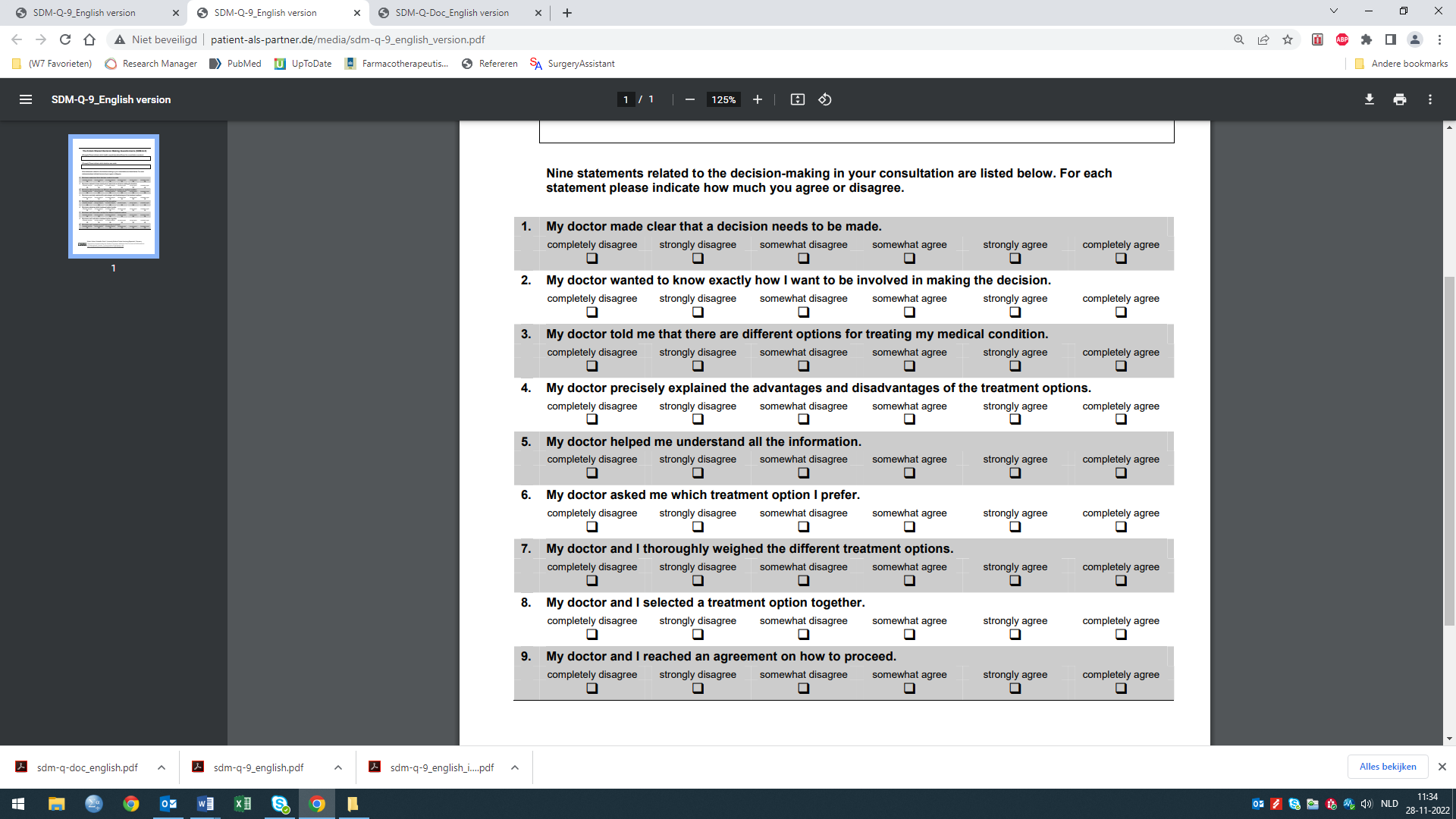


2. My doctor wanted to know exactly how I want to be involved in making the decision.


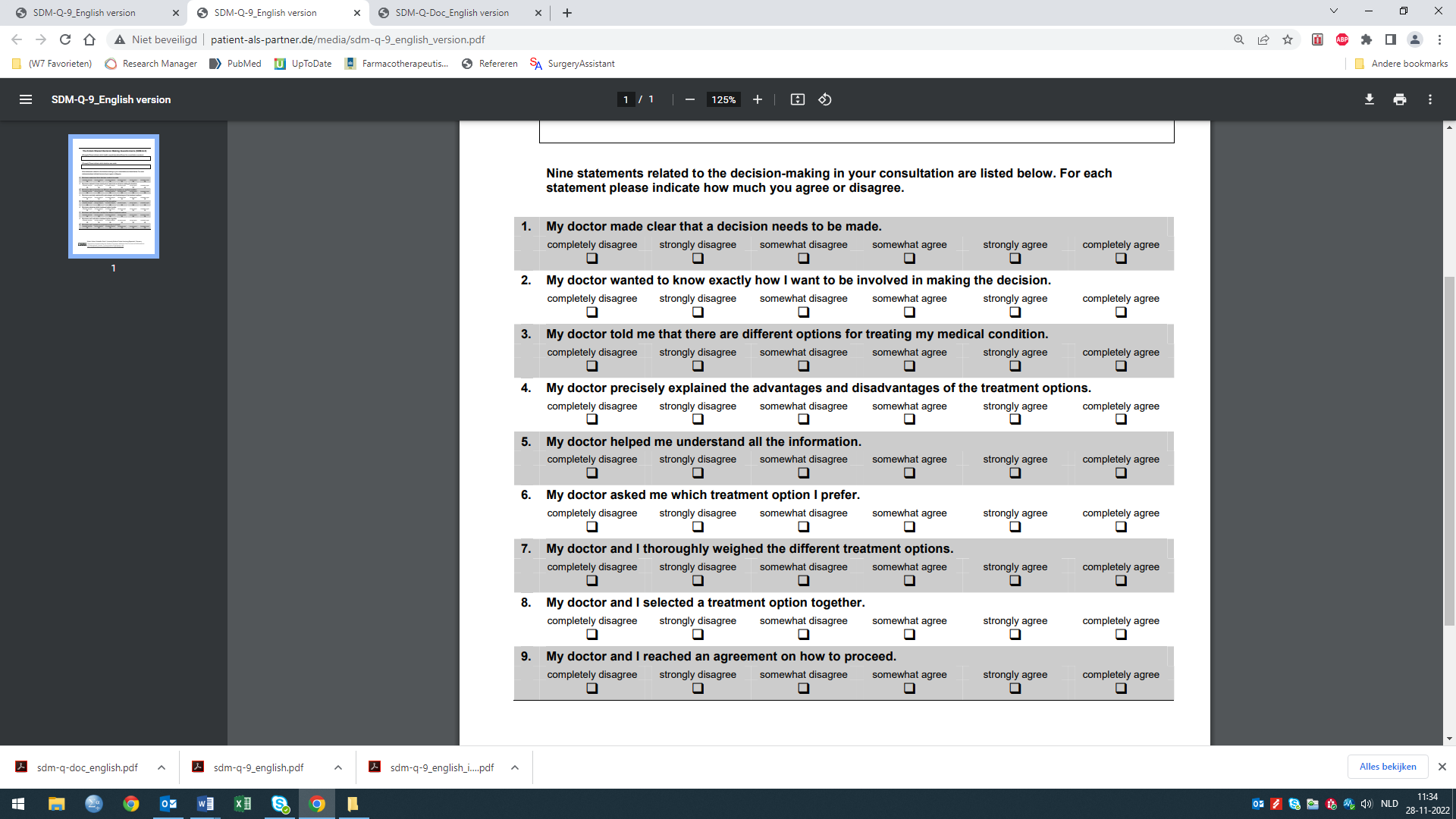


3. My doctor told me that there are different options for treating my medical condition.


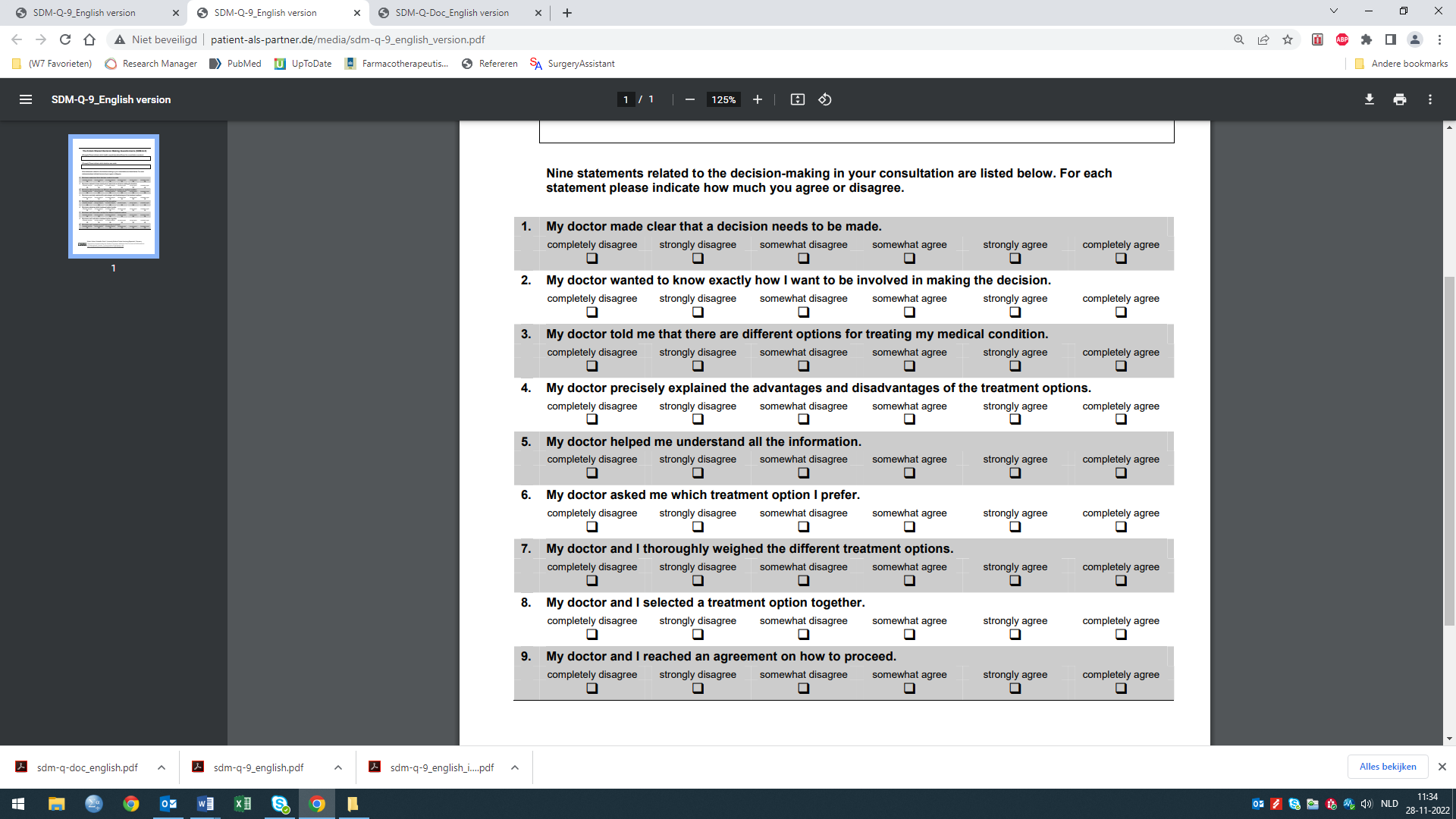


4. My doctor precisely explained the advantages and disadvantages of the treatment options.


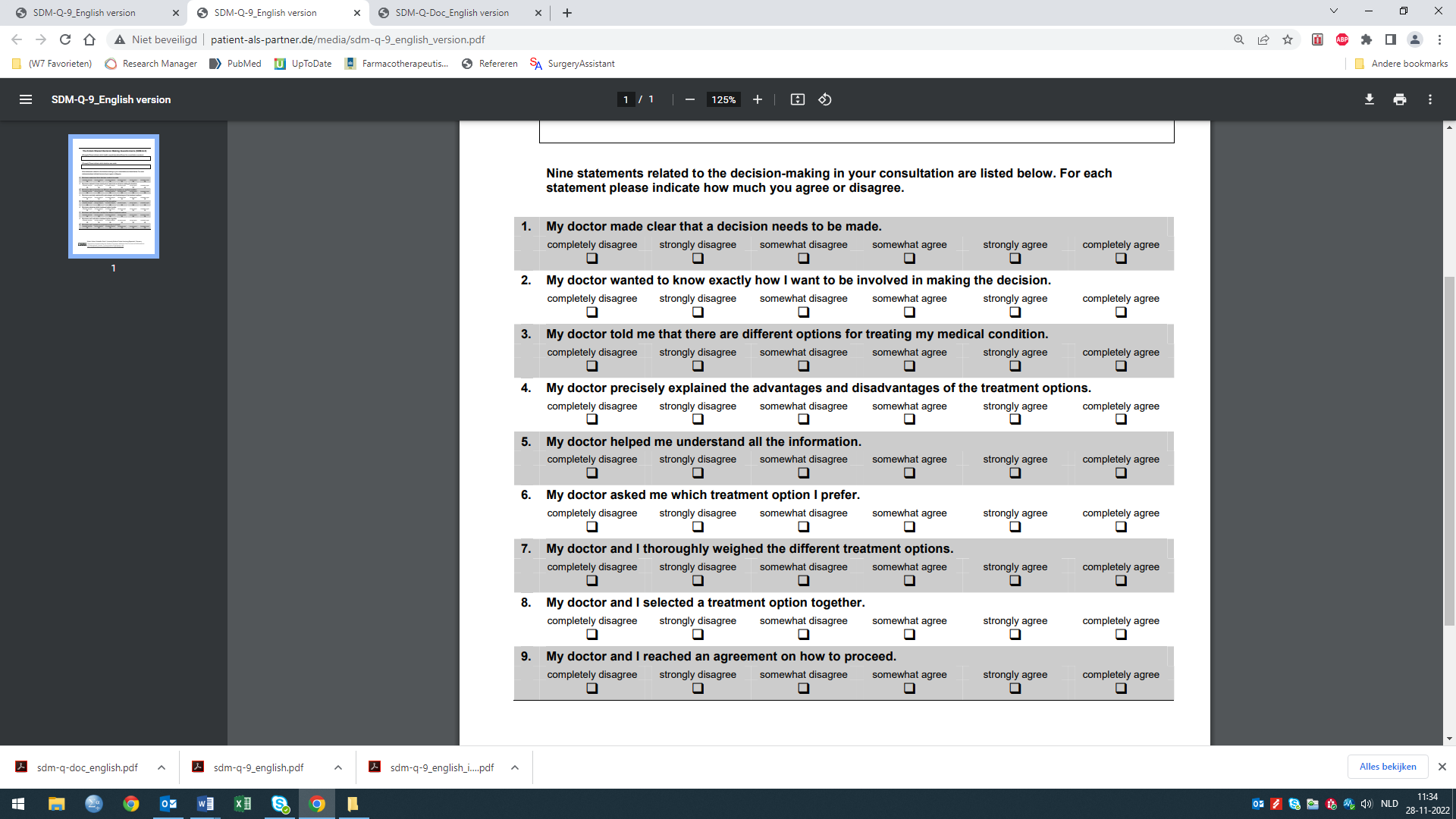


5. My doctor helped me understand all the information.


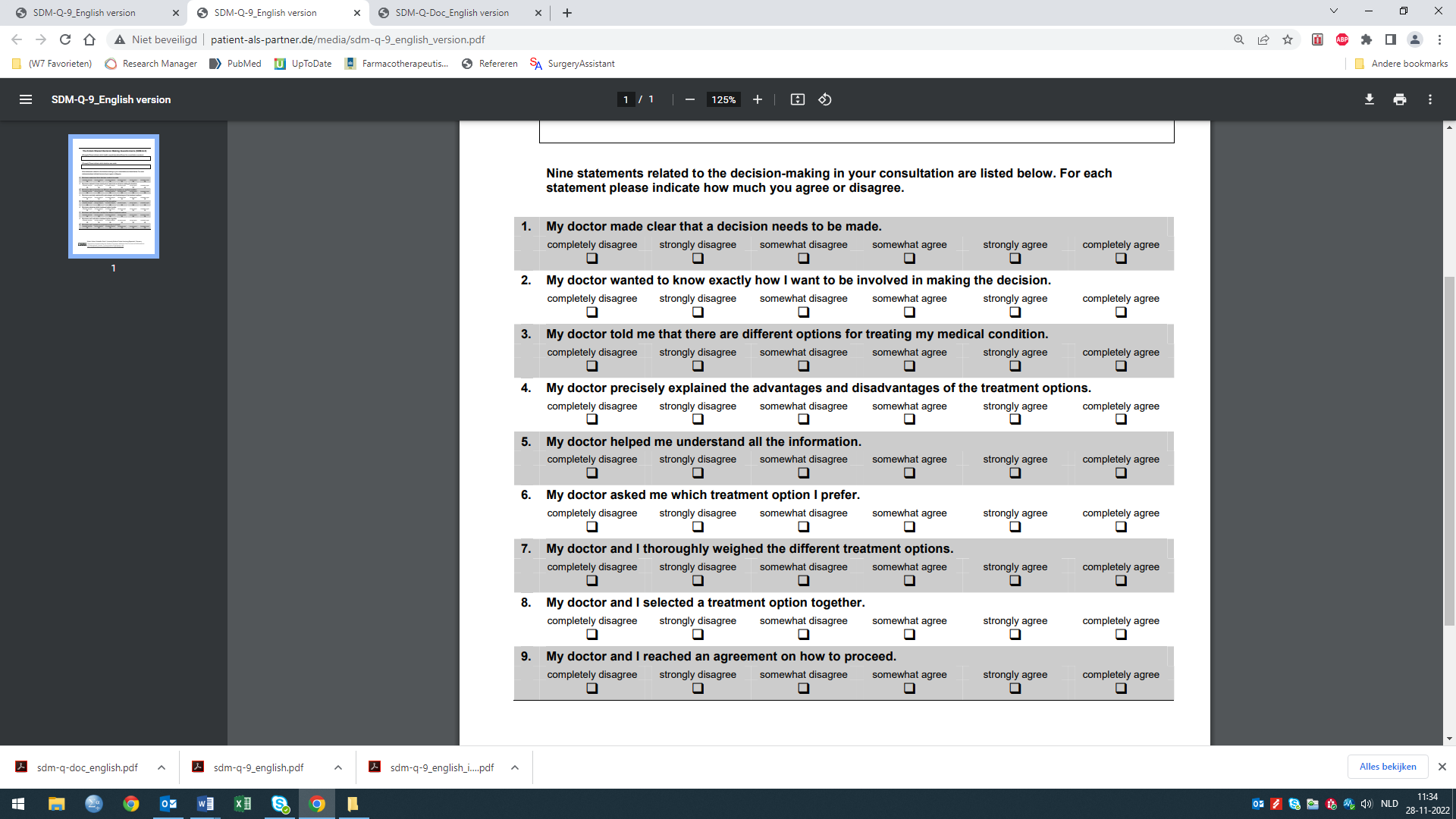


6. My doctor asked me which treatment option I prefer.


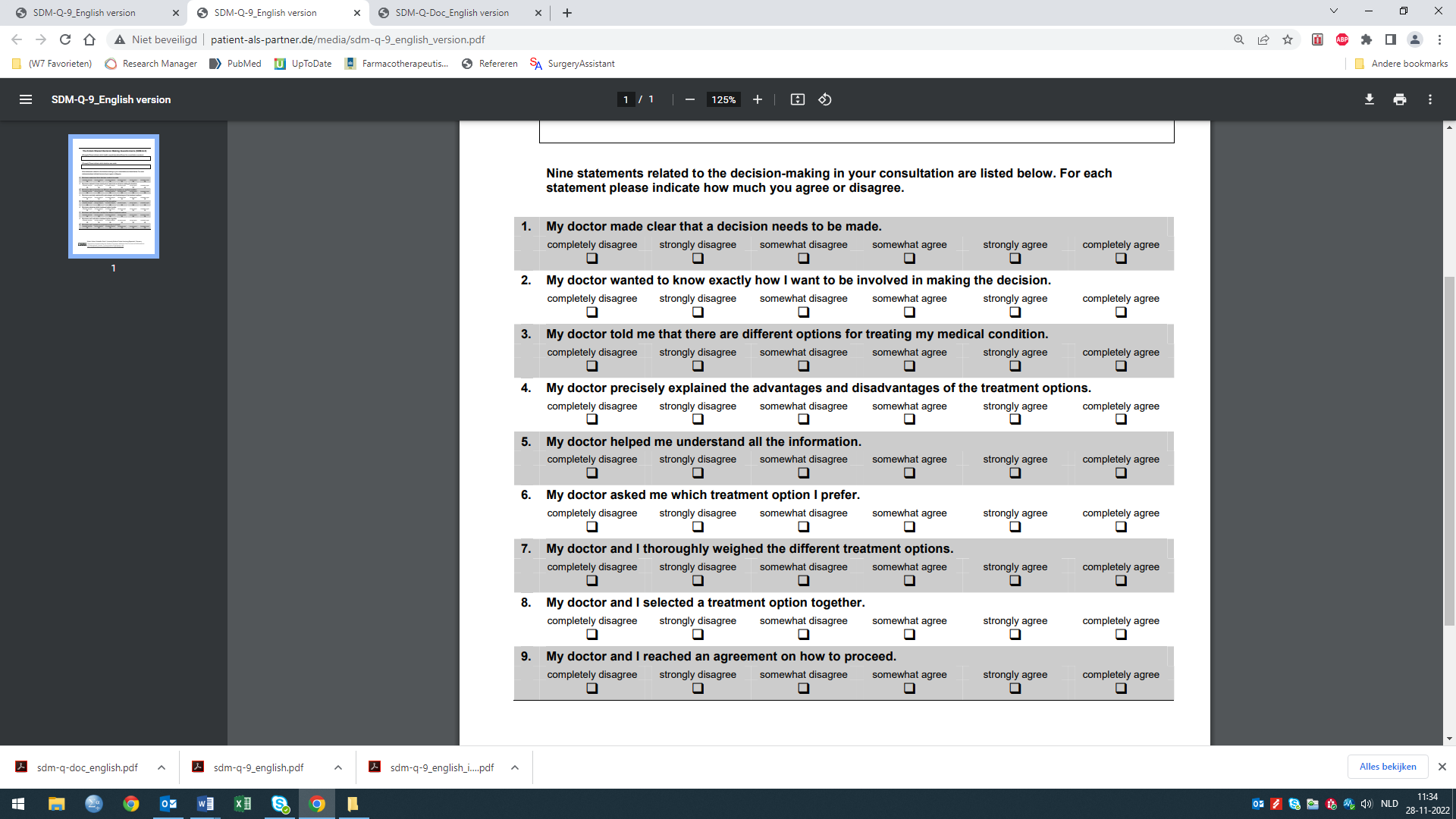


7. My doctor and I thoroughly weighed the different treatment options.


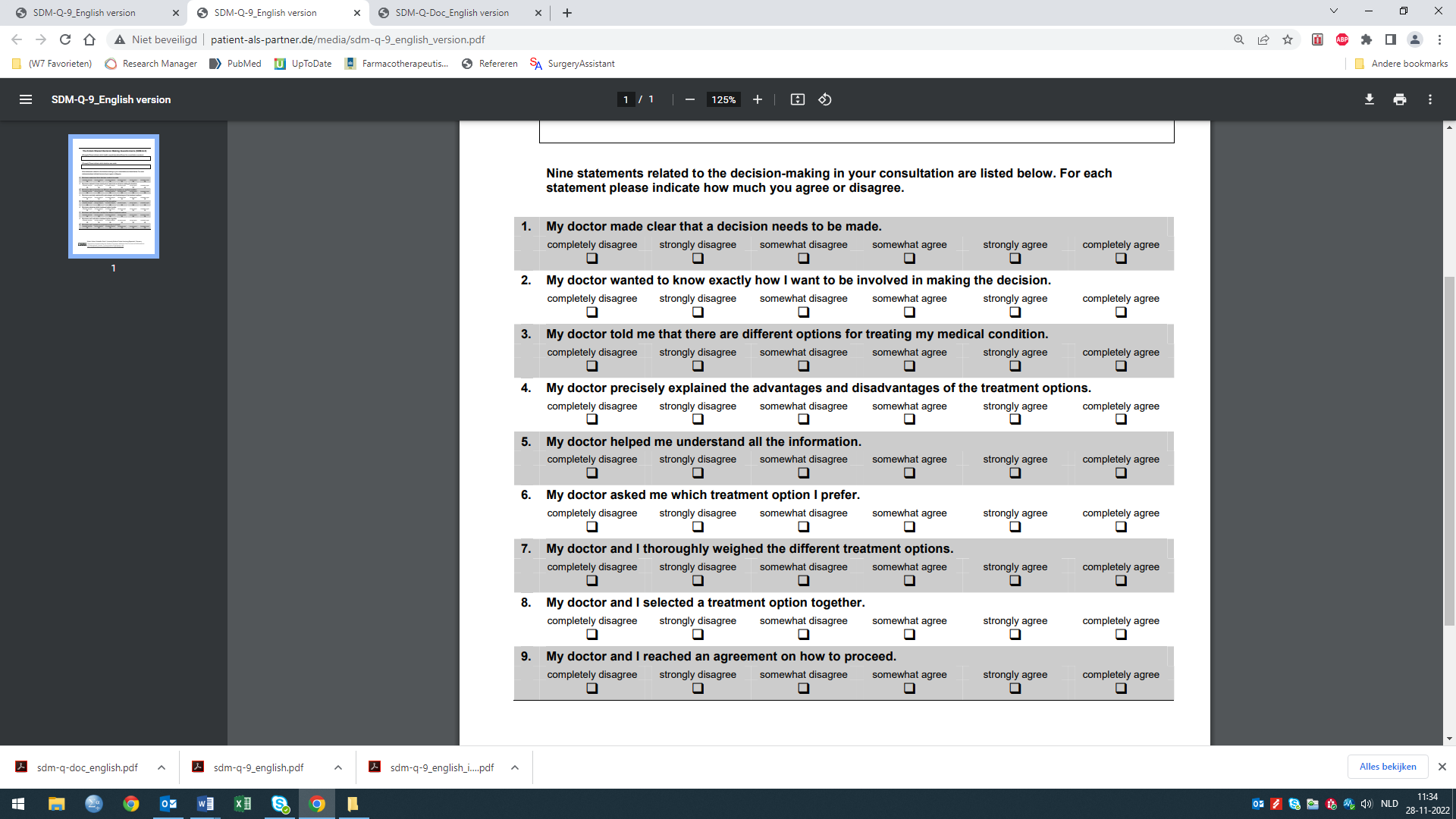


8. My doctor and I selected a treatment option together.


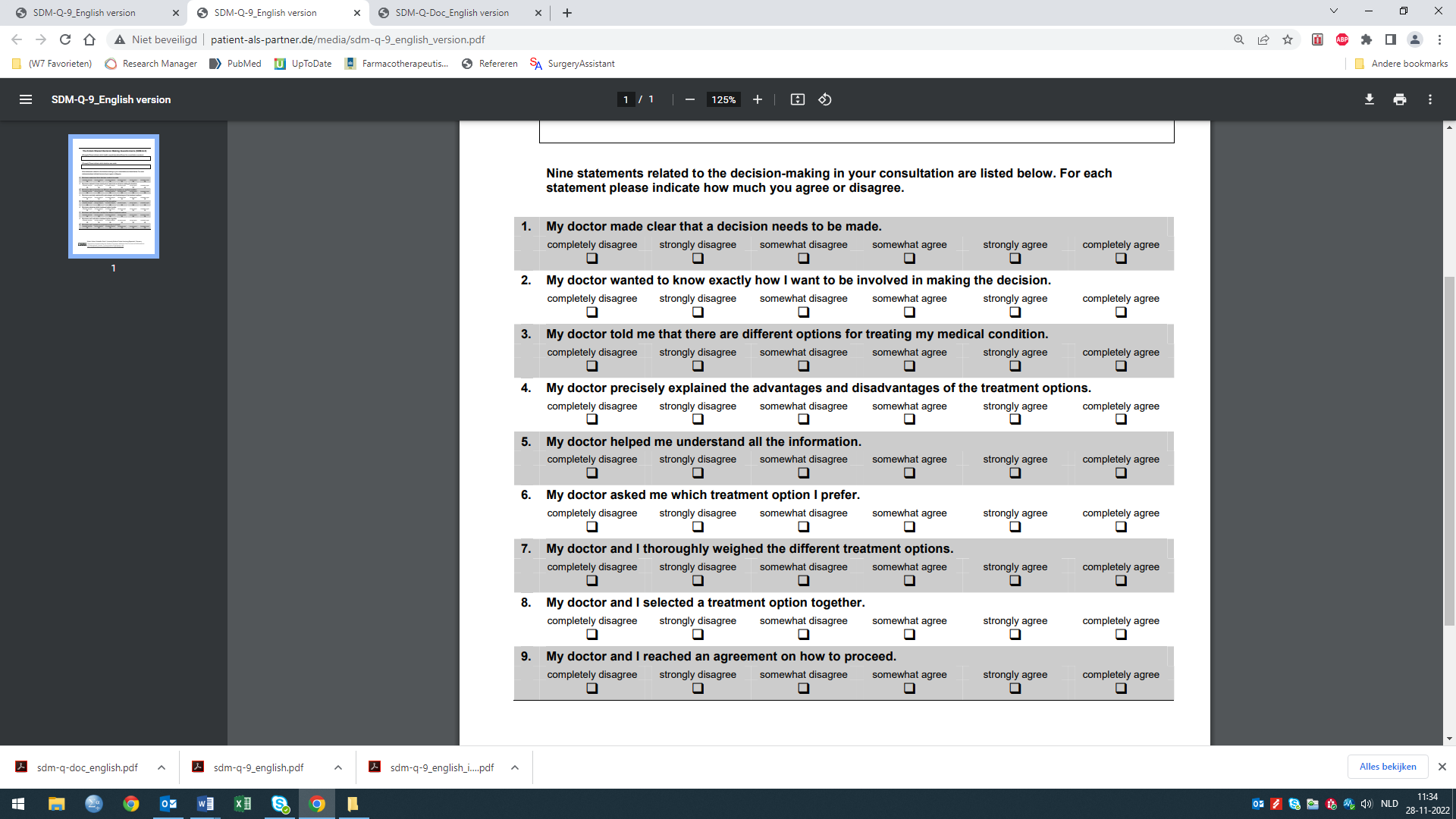


9. My doctor and I reached an agreement on how to proceed.


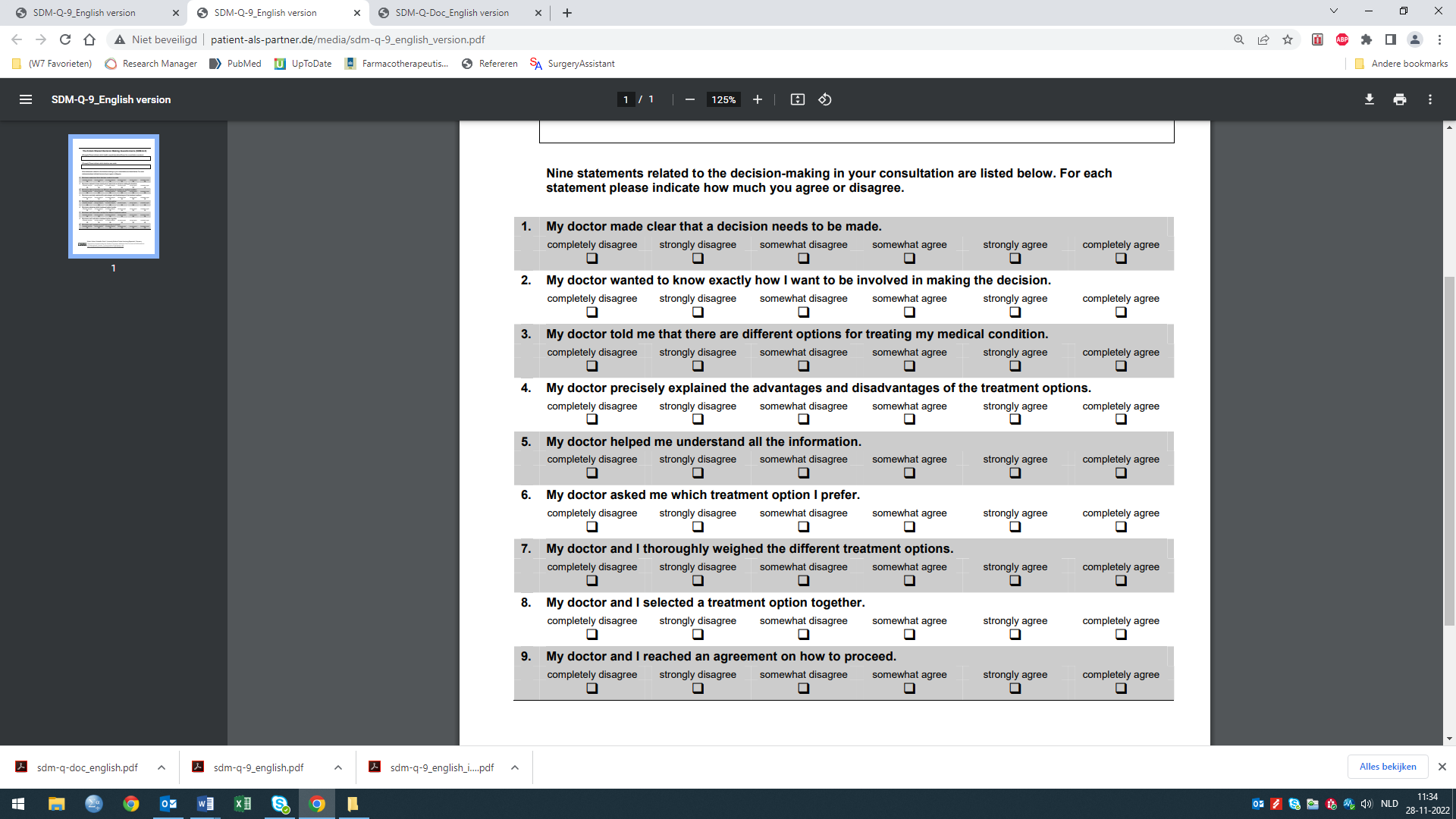


Satisfaction:

On a scale of 0 to 10, how satisfied are you with the decision-making process in this consultation?

*Please choose a number*

Not at all satisfied Extremely satisfied

0 – 1 – 2 – 3 – 4 – 5 – 6 – 7 – 8 – 9 – 10

On a scale of 0 to 10, how likely would you be to recommend this doctor to a friend or colleague?

*Please choose a number*

Not at all likely Extremely likely

0 – 1 – 2 – 3 – 4 – 5 – 6 – 7 – 8 – 9 – 10

**Appendix 3** Physician questionnaire after the consultation

1. Which of the following forms of decision-making did you use regarding the choice for the treatment of your patient?

*Please choose a letter*

| **A** | The patient made the final treatment decision about which treatment she receives |
| --- | --- |
| **B** | The patient made the final decision of the treatment after seriously considering my opinion |
| **C** | The patient and I shared responsibility for deciding which treatment is best for her |
| **D** | I made the final decision about which treatment will be used, but seriously considered the patient’s opinion |
| **E** | I made all decisions regarding the treatment of the patient |

2. Nine statements related to the decision-making in your consultation are listed below.

*For each statement please indicate how much you agree or disagree.*

2.1 I made clear to my patient that a decision needs to be made.


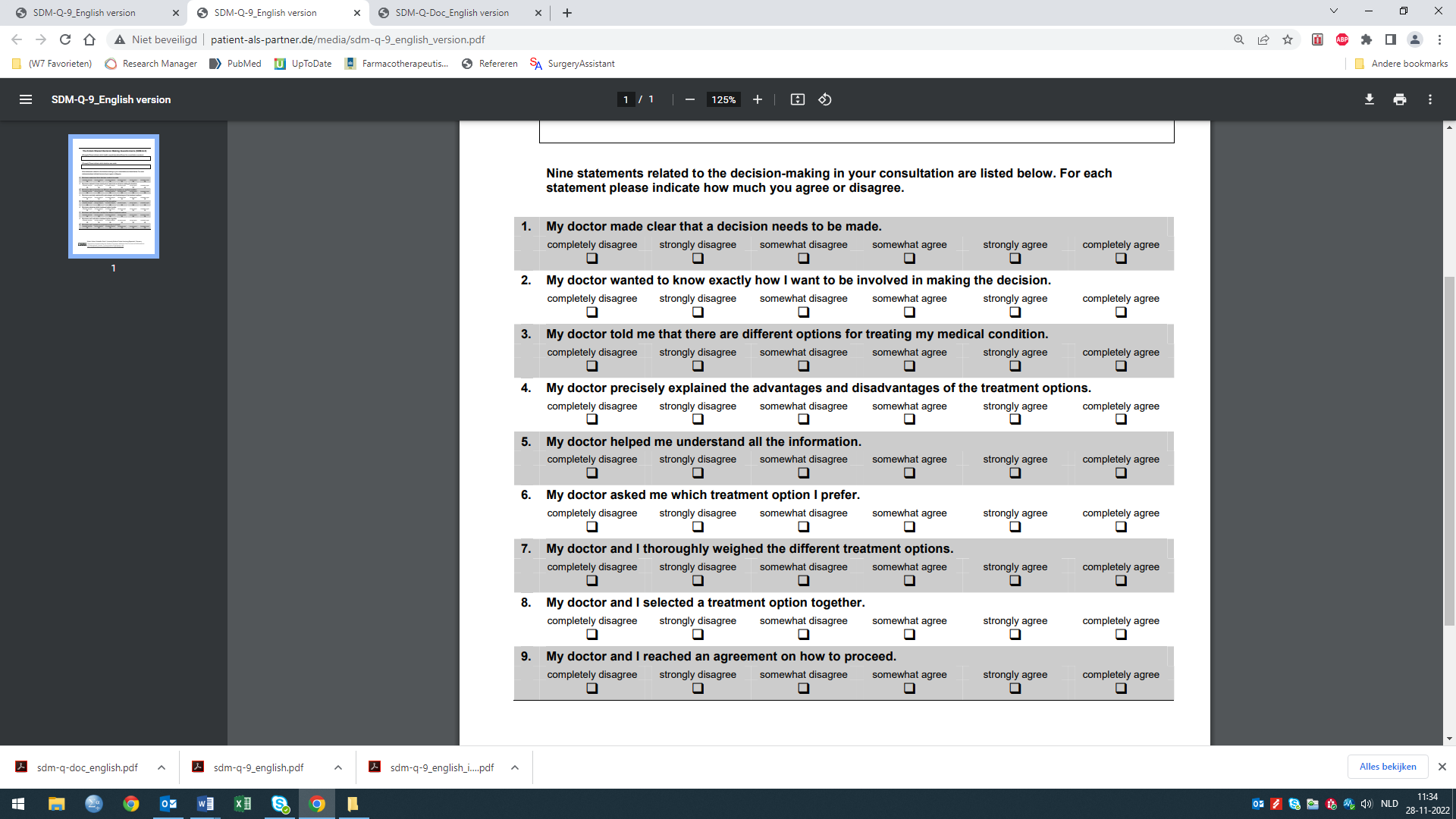


2.2 I wanted to know exactly from my patient how he/she wants to be involved in making the decision.


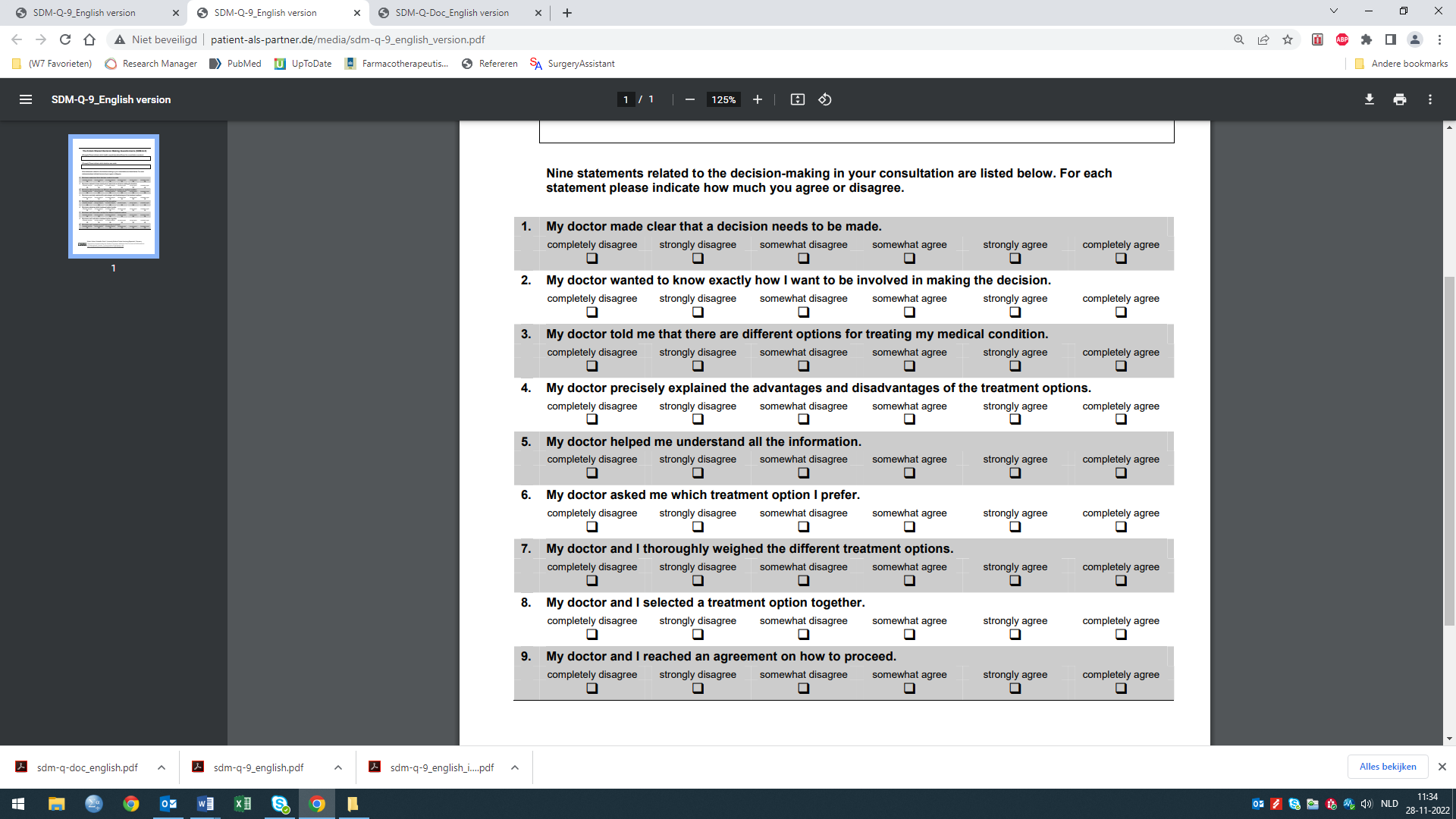


2.3 I told my patient that there are different options for treating his/her medical condition.


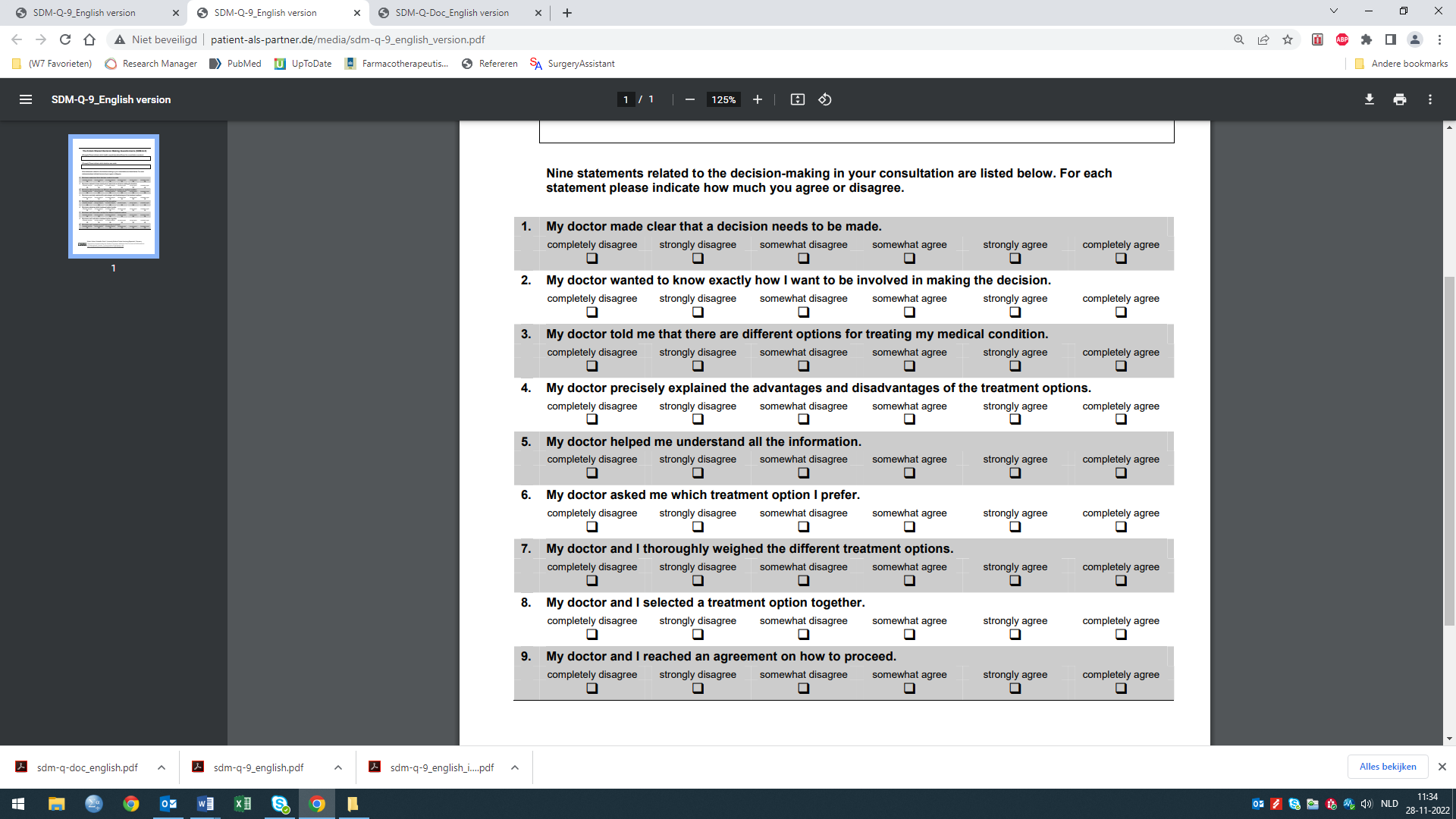


2.4 I precisely explained the advantages and disadvantages of the treatment options to my patient.


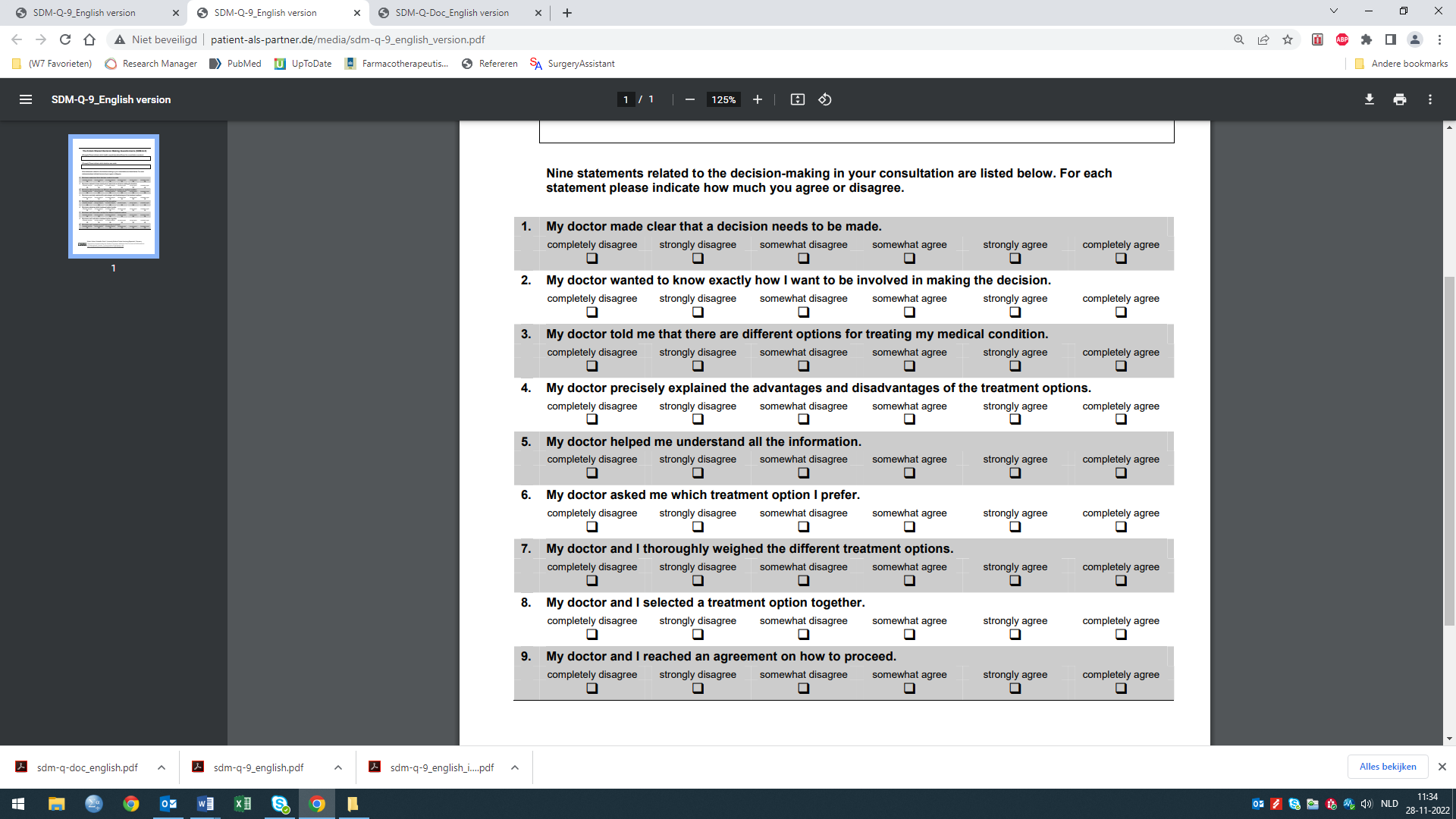


2.5 I helped my patient understand all the information.


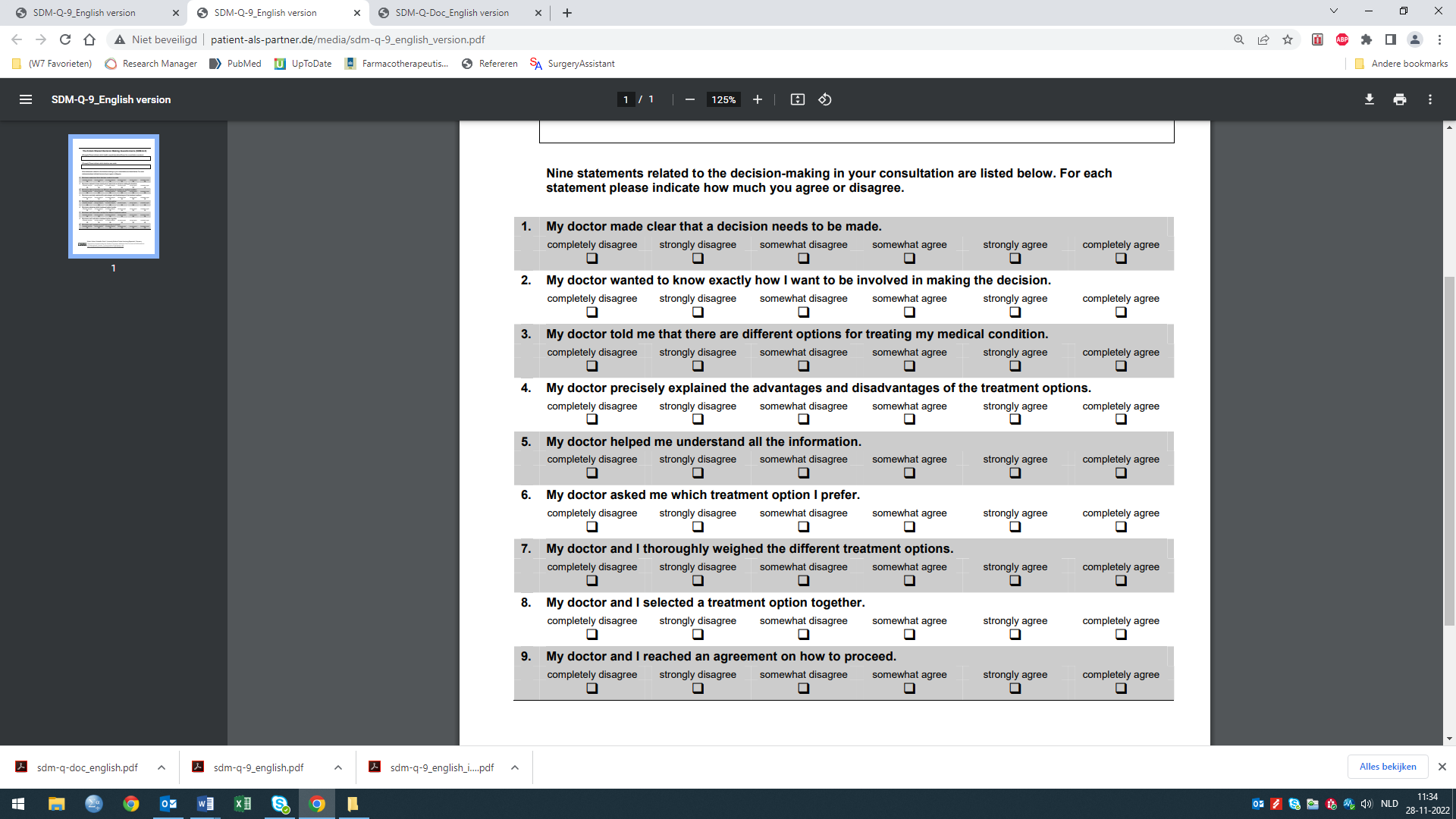


2.6 I asked my patient which treatment option he/she prefers.
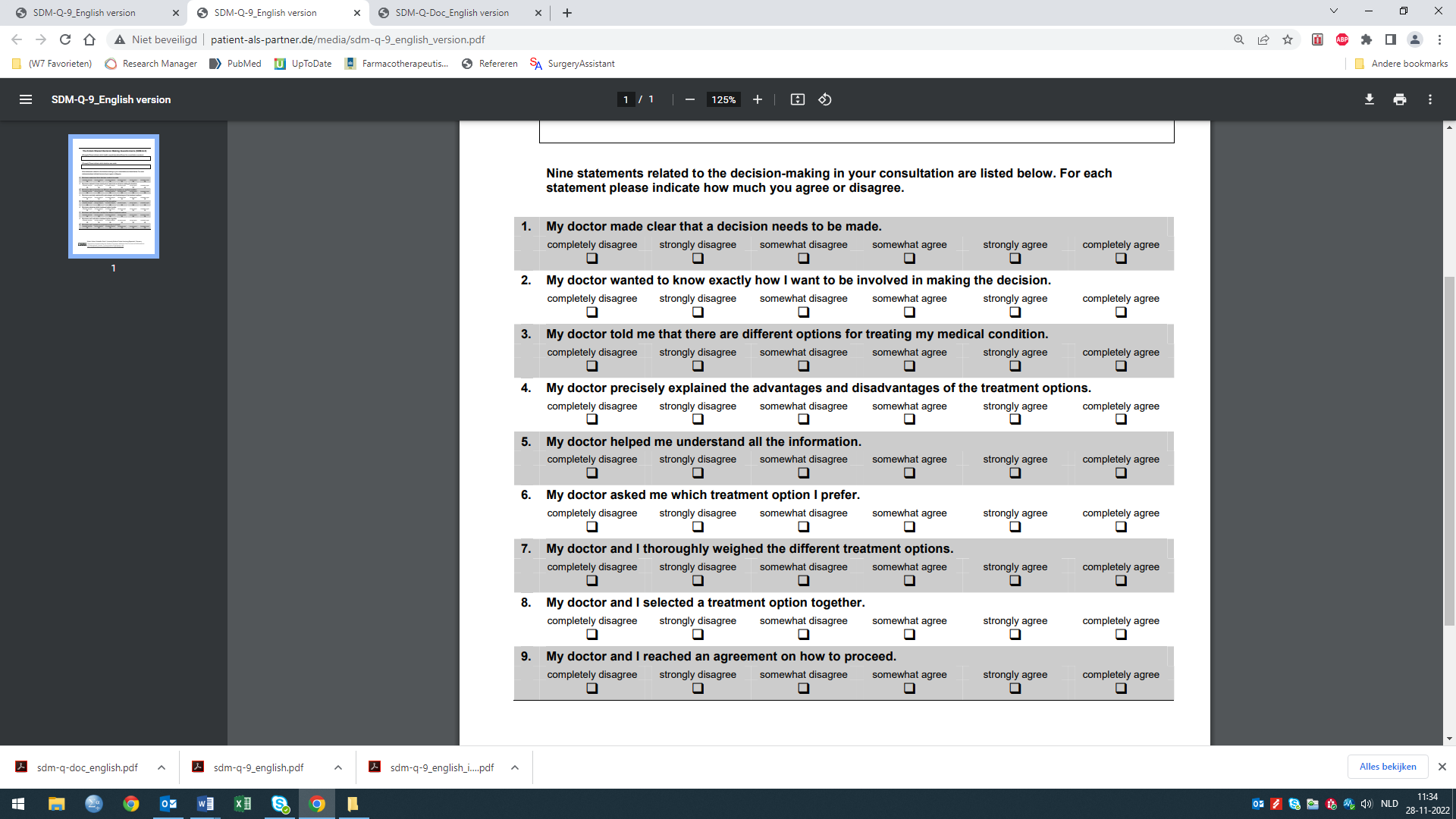


2.7 My patient and I thoroughly weighed the different treatment options.
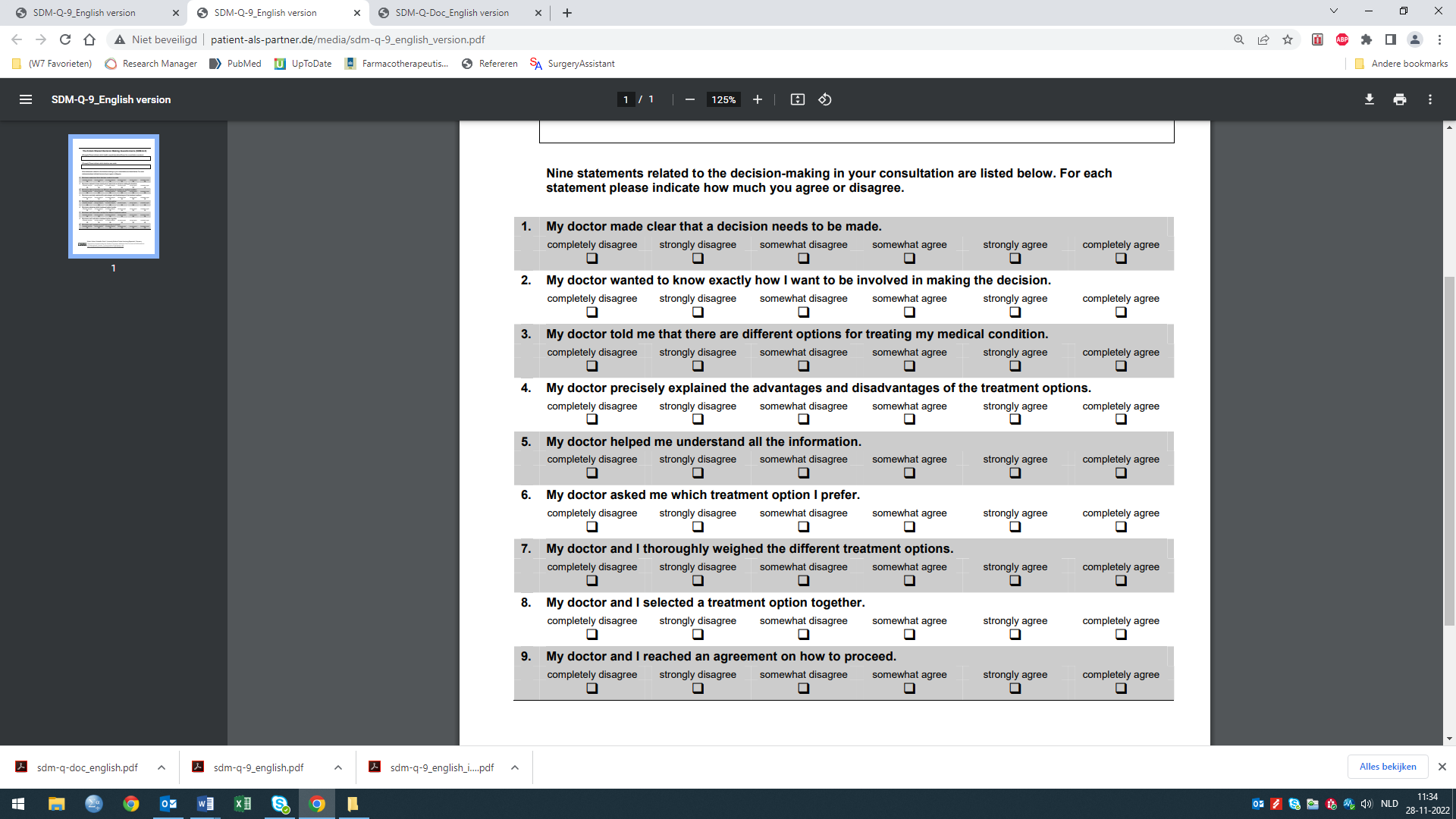


2.8 My patient and I selected a treatment option together.
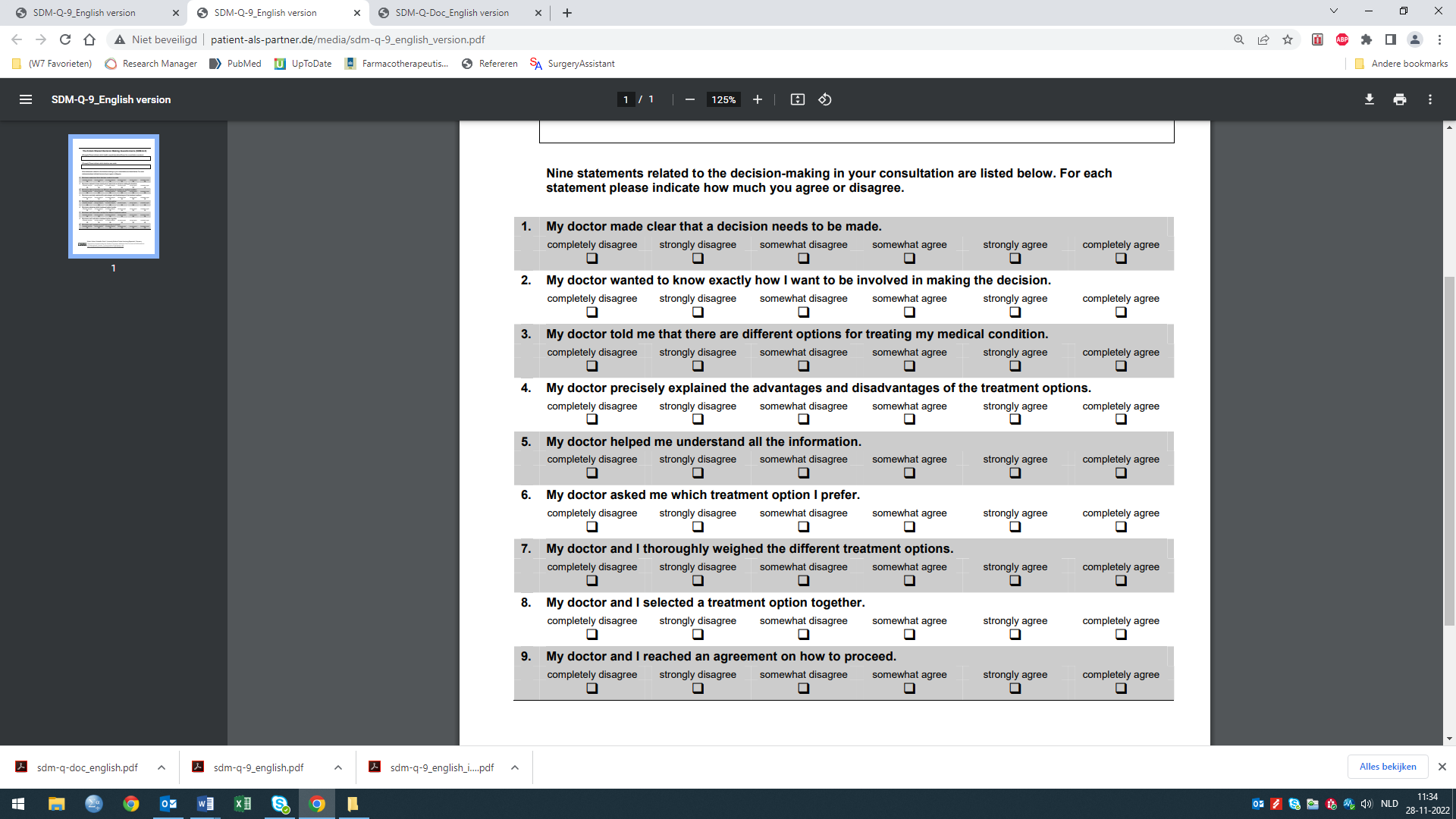


2.9 My patient and I reached an agreement on how to proceed.


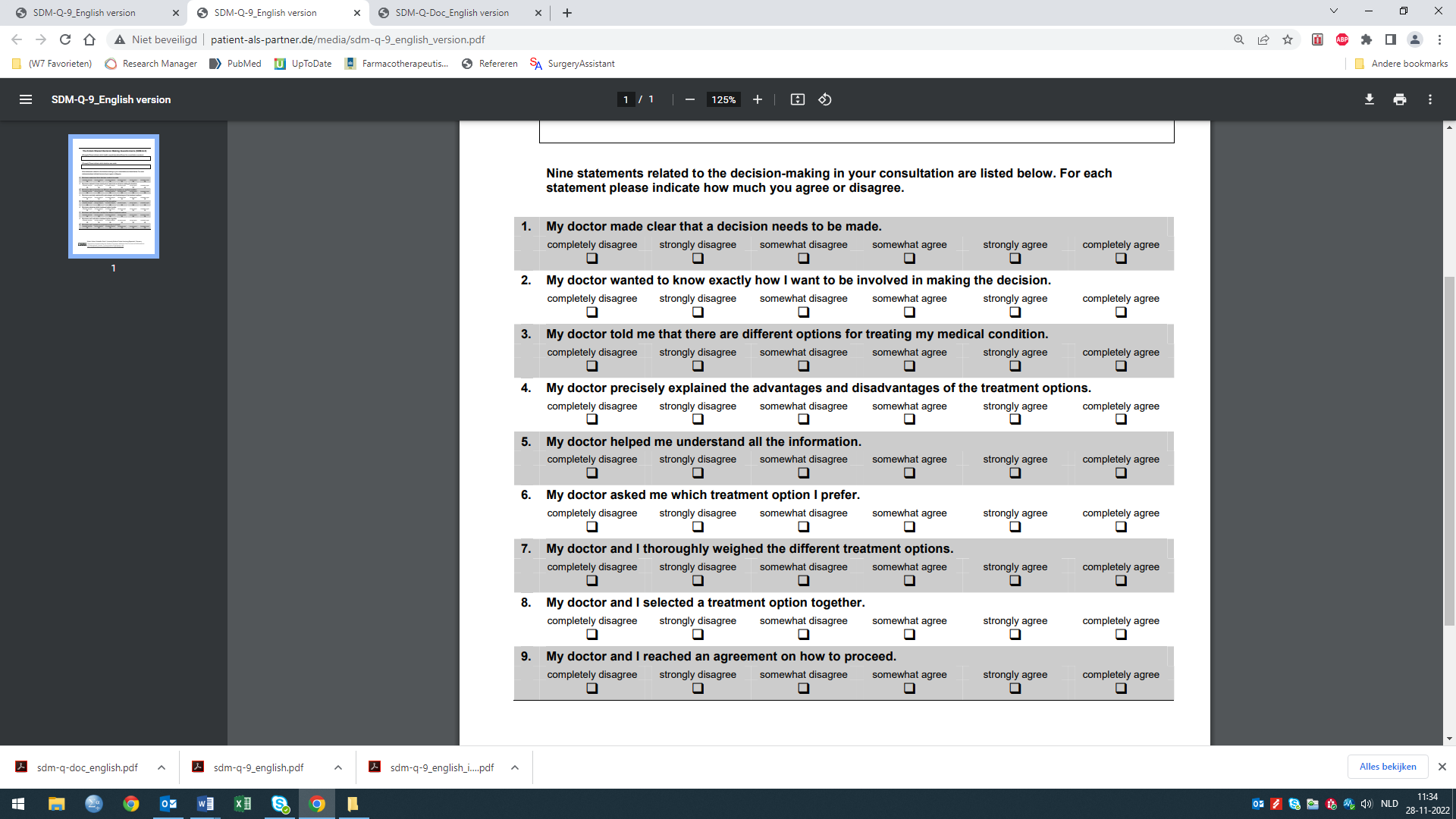


**Appendix 4** OPTION-5

| **OPTION 5-item observation list** | | **Scores** |
| --- | --- | --- |
| 1. | The provider draws attention to, or re-affirms, a problem where alternate treatment or management options exist, and which requires the initiation of a decision making process. If the patient draws attention to the availability of options, and the provider responds by agreeing that the options need consideration, the item can also be scored positively. | 0—not observed  1—problem definition  2—listing the options  3—equality of the options  4—is it clear/any questions |
| 2. | The provider reassures the patient, or re-affirms, that the provider will support the patient to  become informed. The provider will support/explain the need to deliberate about the options. | 0—not observed  1—decide together  2—mention is it a difficult choice  3—will support irrespective of the choice of the patient  4—both options are o.k., depends on the preferences of the patient, provider has a supportive role |
| 3. | The provider gives information, or re-affirms/checks understanding, about options that are considered reasonable (including taking ‘no action’), to support the patient in understanding/comparing the pros and cons. | 0—no information  1—listing the options  2—explaining pros and cons  3—is it clear/any questions  4—ask the patient to repeat the information |
| 4. | The provider supports the patient to examine, voice, and explore his/her personal preference in  response to the options that have been described. | 0—not observed  1—exploring preferences  2—exploring concerns  3—exploring expectations  4—integrates preferences/concerns/expectations for  Recommendation |
| 5. | The provider makes an effort to integrate the patient’s preferences as decisions are either made by the patient or arrives at by a process of collaboration and discussion. | 0—not observed  1—indicates need for decision  2—additional information to review the decision at home  3—appointment for evaluating the decision  4—provider indicates that the patient can abandon earlier choice |
